# Aggregate-selective removal of pathological tau via clustering-activated degraders

**DOI:** 10.1126/science.adp5186

**Published:** 2024-08-29

**Authors:** Jonathan Benn, Shi Cheng, Sophie Keeling, Annabel E Smith, Marina J Vaysburd, Dorothea Böken, Lauren VC Miller, Taxiarchis Katsinelos, Catarina Franco, Elian Dupré, Clément Danis, Isabelle Landrieu, Luc Buée, David Klenerman, Leo C James, William A McEwan

**Affiliations:** 1 UK Dementia Research Institute at the University of Cambridge, Hills Road, Cambridge, CB2 0AH, UK; 2 MRC Laboratory of Molecular Biology, Cambridge CB2 0QH, UK; 3 CNRS EMR9002 – BSI - Integrative Structural Biology, LabEx DISTALZ, F-59000 Lille, France.; 4 Univ. Lille, Inserm, CHU Lille, Institut Pasteur de Lille, U1167 - RID-AGE - Risk Factors and Molecular Determinants of Aging-Related Diseases, F-59000 Lille, France; 5 Université Lille, Inserm, CHU Lille, Lille Neuroscience & Cognition, LabEx DISTALZ, F-59000 Lille, France

## Abstract

Selective degradation of pathological protein aggregates while sparing monomeric forms is of major therapeutic interest. The E3 ligase TRIM21 degrades antibody-bound proteins in an assembly state-specific manner, owing to the requirement of TRIM21 RING domain clustering for activation, yet effective targeting of intracellular assemblies remains challenging. Here, we fused the RING domain of TRIM21 to a target-specific nanobody to create intracellularly expressed constructs capable of selectively degrading assembled proteins. We evaluated this approach against histone 2B-GFP and tau, a protein that undergoes pathological aggregation in Alzheimer’s and other neurodegenerative diseases. RING-nanobody degraders prevented or reversed tau aggregation in culture and in vivo, with minimal impact on monomeric tau. This approach may have therapeutic potential for the many disorders driven by intracellular protein aggregation.

The transition of soluble physiological proteins into pathological homotypic aggregates underlies many common neurodegenerative diseases. Tau is one such protein, whose aggregation correlates with symptomatic progression in Alzheimer’s disease and other neurodegenerative disorders (1, 2) and is therefore a target of therapeutic interest. Antisense oligonucleotides (ASOs) can reduce tau protein accumulation, ameliorating tau pathology in mice (3), and have now entered human trials (4, 5). However, such nucleic acid approaches rely on reducing total protein pools and so loss of physiological protein is unavoidable and potentially problematic. Proteolysis targeting chimeras (PROTACs) directed against tau (6, 7), and previously described intracellularly expressed antibody-based tau degraders (8), are either non-selective or must rely on target binding specificity to encode aggregate selectivity. Methods that potently and selectively deplete aggregated proteins are therefore required.

The cytosolic antibody receptor and E3 ubiquitin ligase TRIM21 can mediate the destruction of antibody-bound targets including viruses (9), cellular proteins (10) and tau aggregates (11). This E3 ligase activity is induced via the clustering and cross-activation of TRIM21’s RING domains (12, 13), which is satisfied upon TRIM21 binding to polyvalent antibody-coated complexes (14) (Fig. 1A and B), resulting in destruction of the entire complex via the unfoldase p97/VCP and the proteasome (15, 16). However, the applicability of targeted protein degradation via this pathway is limited by the general inability of antibodies to access the intracellular domain. For instance, anti-tau immunotherapy can be dependent on TRIM21, but relies on extracellular “seed competent” tau aggregates importing antibodies into the cytosol where they engage and activate TRIM21 to neutralise the bound aggregates (17).

In this study we sought to exploit the clustering-dependent activation of TRIM21 to develop genetically encoded degraders that are activated in the context of assembled, but not monomeric, targets (Fig. 1C and D). By fusing the RING domain of TRIM21 to a target-specific nanobody we generated RING-nanobody (R-Nb) degraders that can be expressed in the nucelocytoplasm. Using tau as a substrate, R-Nb constructs were demonstrated to inhibit seeded tau aggregation and degrade pre-existing insoluble aggregates, whilst leaving soluble monomeric tau largely unaffected.

## Tau targeted R-Nb rapidly degrades aggregated tau species

The repetitive nature of fibrillar tau aggregates was hypothesized to allow for the dense clustering of R-Nb degraders on their surface, inducing RING cross-activation and subsequent degradation. A variant of the anti-tau C-terminus nanobody F8-2, optimised for increased intracellular affinity (S54L+T127A), (18), was selected as a targeting domain (R-Nb_F8-2_). This construct consisted of an N-terminal TRIM21 RING domain followed by the nanobody and a self-cleaving (T2A) mCherry reporter (Fig. S1A). To create a cell line model containing constitutively aggregated fluorescently tagged tau, HEK293 cells over-expressing soluble P301S tau-venus (11) (tau-venus soluble, TVS cells) were seeded with tau assemblies prepared from the brains of aged Tg2541 P301S tau transgenic mice (19) and clonally expanded (tau-venus aggregated, TVA cells). Immuno-gold electron microscopy confirmed the presence of fibrillar tau assemblies in TVA cells (Fig. S1B and C).

**Fig. 1:**
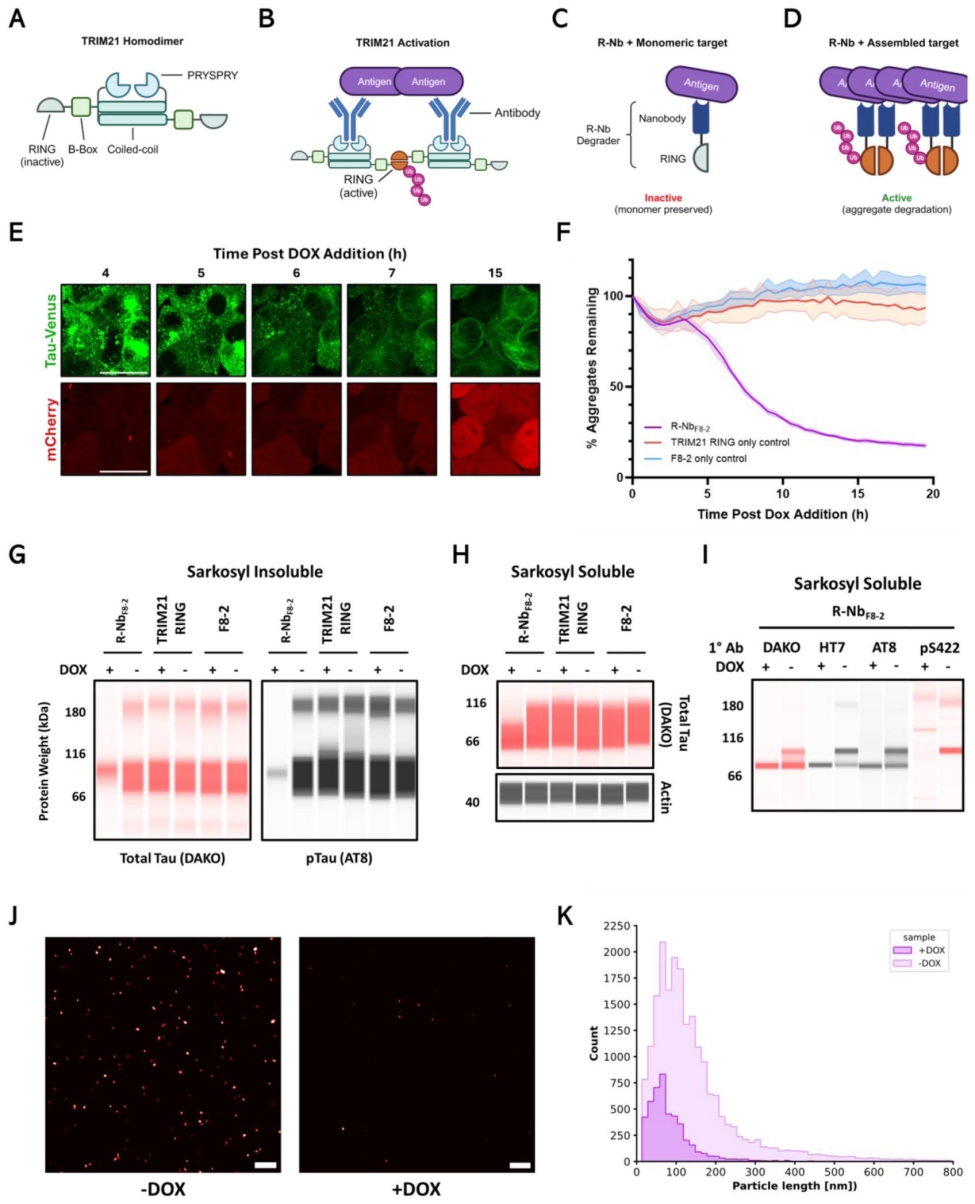
R-Nb_F8-2_ rapidly degrades aggregated tau species. (**A**) Cytosolic TRIM21 exists as an inactive homodimer, with each monomer consisting of a RING E3 ligase domain, B-Box, Coiled-coil, and an antibody Fc binding PRYSPRY domain. (**B**) The clustering of TRIM21 upon antibody-coated polyvalent substrates allows for RING E3 ligase domain cross-activation and subsequent polyubiquitination. (**C, D**) TRIM21 RING-Nanobody (R-Nb) degraders were engineered to similarly require RING domain cross-activation to induce target degradation and therefore become active upon binding to (D) homotypic assemblies of antigens but not (C) monomeric antigens. (**E**) Live cell fluorescent microscopy of TVA cells which express aggregated P301S tau-venus and DOX-inducible degrader, R-Nb_F8-2_, with an mCherry reporter. Scale bar, 20µm. (**F**) Quantification of tau-venus aggregates from live cell imaging of TVA cells expressing DOX inducible R-Nb_F8-2_, TRIM21 RING, or F8-2. Images were captured and analyzed every 30 minutes for 20 hours following DOX addition. (**G**) Capillary based immunoblots of sarkosyl insoluble fractions from TVA cell lines as above treated with or without DOX for 15 hours. Samples were probed for total tau (DAKO) and phospho-tau (pTau) (AT8). (**H**) Sarkosyl soluble fractions from the same experiment were probed for total tau (DAKO) and actin as a loading control. (**I**) Sarkosyl soluble samples were further analyzed by diluting 1:4 in PBS and probing with total tau (DAKO and HT7) and pTau (AT8 and pS422) antibodies. (**J**) Representative super-resolution microscopy images of tau-venus aggregates in lysates from TVA cells expressing DOX inducible R-Nb_F8-2_ treated with or without DOX for 15 hours, using anti-tau antibody HT7 for both capture and detection. Scale bar, 1µm (**K**) Histogram showing the size and count of tau-venus aggregates detected from super-resolution microscopy images. Shaded areas represent mean ± SD. (F) n = 8 biological replicates per condition. (K) n = 5 or 6 technical replicates per condition.

To assess the ability of R-Nb_F8-2_ to degrade pre-formed tau-venus aggregates, TVA cells were modified to express R-Nb_F8-2_, or controls, in a doxycycline (DOX) inducible manner. Addition of DOX resulted in engagement of tau aggregates by the nanobody and a rapid reduction of punctate tau-venus aggregates, concomitant with R-Nb_F8-2_ and mCherry expression (Fig. 1E and F, Supp. Video 1, Fig S1D-F). 15 hours post-DOX addition, tau-venus appeared re-solubilised and microtubule-associated. TRIM21 RING or F8-2 alone did not substantially alter the number of tau-venus aggregates, despite comparable construct expression (Fig. S1D).

To probe the nature of tau-venus aggregates degraded by R-Nb_F8-2_, the sarkosyl soluble and insoluble fractions from these cells following DOX treatment were examined. As expected, a reduction in insoluble total and phosphorylated (AT8, pSer202 & pThr205) tau was observed only in R-Nb_F8-2_ expressing cells (Fig. 1G). Biochemically soluble tau species were also partially reduced following R-Nb_F8-2_ expression (Fig. 1H). Subsequent probing revealed that the depleted species ran as a distinct higher molecular weight band and were recognised by phospho-tau antibodies (Fig. 1I), therefore likely representing small hyperphosphorylated tau assemblies such as have previously been reported (20). Further interrogation of tau species remaining in TVA lysates was performed using single-molecule pull-down (SiMPull) super-resolution microscopy (21), revealing that tau assemblies of all sizes were reduced following R-Nb_F8-2_ expression (+DOX) (Fig.1J and K).

## Low affinity R-Nb constructs selectively degrade aggregated tau, including seed competent species

In its physiological state, tau exists as a soluble cytosolic or microtubule-bound protein. We hypothesized that recruitment of R-Nb degraders to soluble tau may not induce degradation due to the spatial separation of RING domains preventing cross-activation. As R-Nb recruitment density will also be driven by nanobody affinity, we further hypothesized this would determine aggregate selectivity. To test this, we assessed a panel of R-Nb constructs with a 1000-fold range of in vitro affinity for tau-venus. Variants of F8-2 were used alongside H3-2 (overlapping epitope with F8-2 (Fig. S2A and B)), with in vitro affinities (K_D_) ranging from ~1.3 µM to 32 nM (18) (Fig. S2C and D). vhhGFP4, which targets venus with an affinity of 1 nM (22) was also used, with a nanobody (Nb139) against an irrelevant target (p53) being used as a negative control (23).

**Fig. 2:**
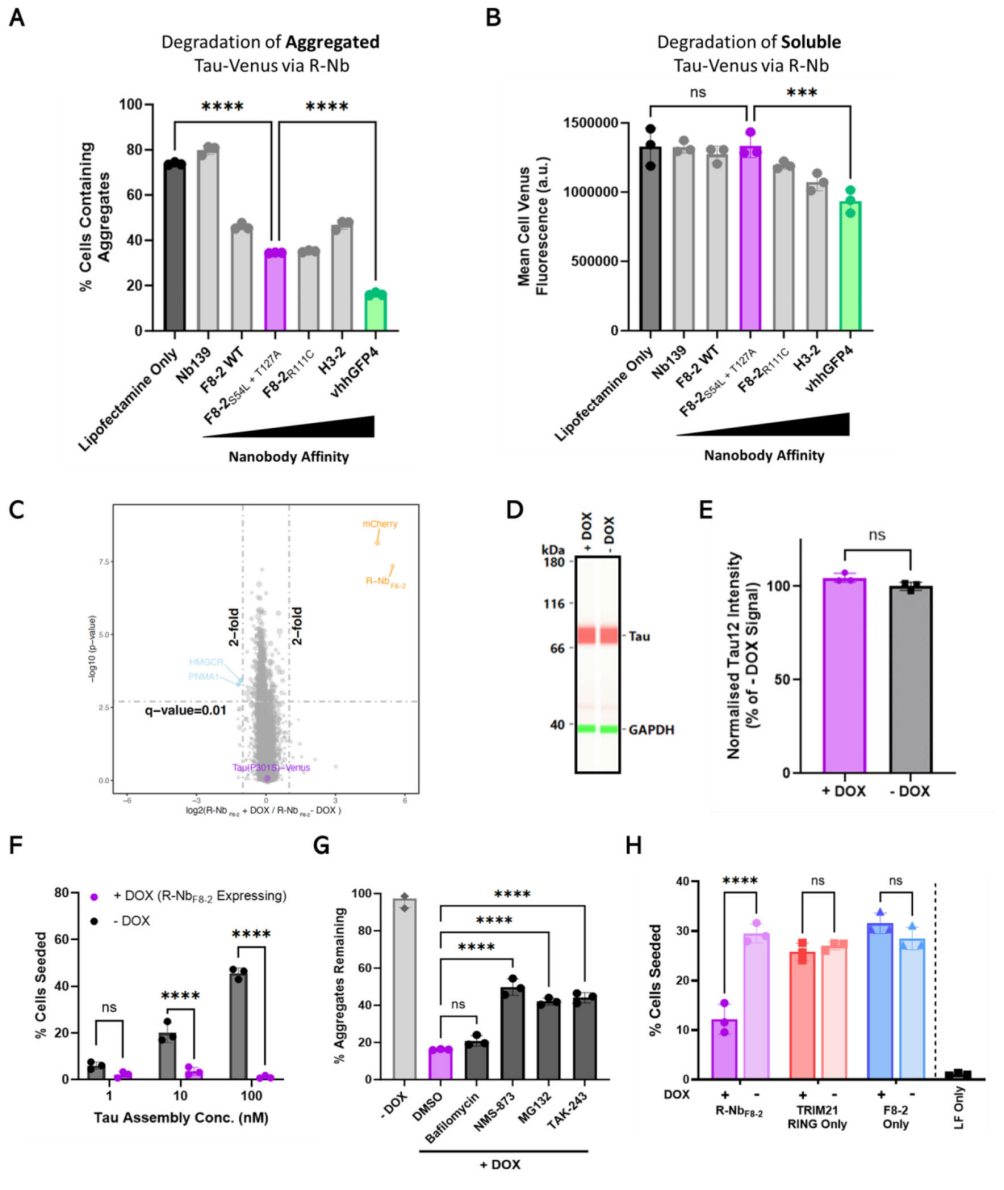
Selective degradation of aggregated tau by R-Nb is dependent on nanobody affinity and protects cells against seeded tau aggregation. (**A**) TVA cells were transfected with R-Nb plasmids employing nanobodies of varying in vitro affinities for tau-venus. F8-2 WT (1332 nM), F8-2_S54L+T127A_ (683 nM), F8-2_R111C_ (211 nM), H3-2 (32 nM), vhhGFP4 (1 nM) or Nb139 (p53 targeting, negative control). 24 hours post-transfection the percentage of cells containing aggregated tau-venus was quantified via fluorescence microscopy. (**B**) TVS cells were transfected with the same R-Nb plasmids, with venus fluorescence quantified by flow-cytometry 24 hours post-transfection. (**C**) DOX inducible R-Nb_F8-2_ TVA cells were lysed 15 hours post-DOX addition, and their proteome compared to untreated (-DOX) cells via mass spectrometry. Significantly altered proteins (q-value <0.01, fold change >2 or <-2) are labeled, alongside TauP301S-Venus. (**D**) Immunoblot of lysates from DOX inducible R-Nb_F8-2_ TVS cells, 24 hours after +/- DOX treatment. Samples probed for tau (Tau12 antibody) and GAPDH. (**E**) Amounts of tau, normalized to GAPDH, in TVS cells + DOX relative to – DOX amounts. (**F**) TVS cells pretreated +/- DOX for 24 hours were challenged with heparin assembled P301S tau assemblies. Percentage of cells containing tau-venus aggregates quantified 72 hours later via fluorescent microscopy. (**G**) Percentage of tau-venus aggregates remaining in TVA cells expressing DOX inducible R-Nb_F8-2_, treated with E1/Proteasome/VCP/autophagy inhibitors (solubilized in DMSO) and DOX for 10 hours, compared to TVA without DOX, quantified by fluorescence microscopy. TAK-243, 100 nM; MG132, 125 nM; NMS-873, 5 µM; Bafilomycin A1, 400 nM. (**H**) Lysates from TVA cells expressing DOX inducible R-Nb_F8-2_, or RING/F8-2 only controls, treated +/- DOX for 15 hours, were introduced to TVS cells via Lipofectamine (LF). Seeded aggregation of tau-venus was quantified 72 hours later via fluorescent microscopy. Error bars indicate mean ± SD. (A), (B), (E), (F), (G) and (H) n = 3 biological replicates. (C) n = 3 technical replicates. (A), (B), (G) and (H) one-way analysis of variance (ANOVA) with Tukey’s correction for multiple comparisons. (E) unpaired *t* test. (F) two-way ANOVA with Šídák’s correction for multiple comparisons. ns, not significant; ***P < 0.001; ****P < 0.0001.

R-Nb constructs were transfected into TVA or TVS cells to assess their ability to degrade aggregated or soluble tau respectively. Super-resolution microscopy has confirmed that P301S tau-venus in TVS cells is localised to microtubules and free in the cytosol (24). All R-Nb constructs were soluble when expressed in HEK293 cells (Fig. S3A), and those capable of binding tau-venus elicited the degradation of aggregates in TVA cells (Fig. 2A). However, degradation of soluble tau-venus in TVS cells was only instigated by R-Nb constructs using high affinity nanobodies (Fig. 2B). All R-Nb constructs were expressed in similar amounts (Fig. S3, B and C). The degradation kinetics of tau-venus were not substantially affected by its aggregation state following expression of the R-Nb_vhhGFP4_ degrader (Fig. S3 D-G). These results demonstrate that degradation may be driven by R-Nb clustering on the surface of fibrillar assemblies for low-affinity degraders, or by high affinity recruitment to more spatially separated targets, and that low affinity R-Nb constructs may permit aggregate-selective degradation. The R-Nb construct using F8-2_S54L+T127A_ (used in Fig. 1) resulted in the highest amounts of aggregate degradation without affecting soluble tau and was therefore selected for use in subsequent experiments (referred to as R-Nb_F8-2_).

To assess the selectivity of R-Nb_F8-2_ degradation the proteome of TVA R-Nb_F8-2_ cells +/- DOX was analysed via mass spectrometry, revealing that out of over 8,000 endogenous proteins quantified, only two (HMGCR and PNMA1) were moderately yet significantly (q-value = 0.01, fold change >2) affected following R-Nb_F8-2_ expression (Fig. 2C). The overall amount of tau-venus protein was unaffected, again suggesting that R-Nb_F8-2_ mediated degradation of aggregated tau is assembly state specific. To confirm that R-Nb_F8-2_ does not degrade soluble tau, TVS cells were modified to express DOX inducible R-Nb_F8-2_. 24 h after DOX addition cells expressed high amounts of R-Nb_F8-2_ (Fig. S4A), yet tau-venus protein remained unaffected (Fig. 2, D and E). Using this same cell line, we tested whether R-Nb_F8-2_ expression would protect against seeded tau aggregation. TVS cells challenged with heparin-assembled P301S tau fibrils in the presence of Lipofectamine form quantifiable aggregated tau-venus puncta in a dose responsive manner (11)**;** this aggregation was almost entirely prevented in TVS cells expressing R-Nb_F8-2_ (Fig. 2F). R-Nb_F8-2_ expression therefore specifically degrades aggregated tau and prevents seeded tau aggregation.

TRIM21 mediated degradation is reliant on the UPS with involvement of the unfoldase p97/VCP (9, 15). To investigate whether degradation of aggregated tau in TVA cells via R-Nb_F8-2_ is reliant on these same mechanisms, we inhibited: 1) the E1 ubiquitin activating enzyme (UAE) with TAK-243; 2) the proteasome with MG132 (Fig. S4B); or 3) VCP with NMS-873 (Fig S4C). Alternatively, bafilomycin was used to inhibit autophagy (Fig. S4D). Solvent only (DMSO) and the inhibitors had minimal effects on R-Nb_F8-2_ expression (Fig. S4E), and DMSO itself did not affect R-Nb_F8-2_ mediated degradation (Fig. S4F). Inhibition of E1, VCP, and the proteasome substantially impaired the ability of R-Nb_F8-2_ to degrade aggregated tau-venus (Fig. 2G). In contrast bafilomycin had no effect, confirming that R-Nb_F8-2_ mediated degradation of aggregated tau, similar to TRIM21, requires the UPS with involvement of VCP.

The degradation of aggregated tau via VCP and the proteasome has been reported to result in production of further seed competent tau species (24, 25). Lysates and supernatants from TVA cells expressing R-Nb_F8-2_ or controls were therefore introduced into TVS cells to induce seeded tau-venus aggregation; only R-Nb_F8-2_ expression reduced the amounts of seed competent tau species (Fig. 2H and S4G). The degradation of aggregated tau via R-Nb_F8-2_ thus reduces total seed competent tau species rather than increasing them.

## In vivo application of R-Nb for CNS targets

**Fig. 3:**
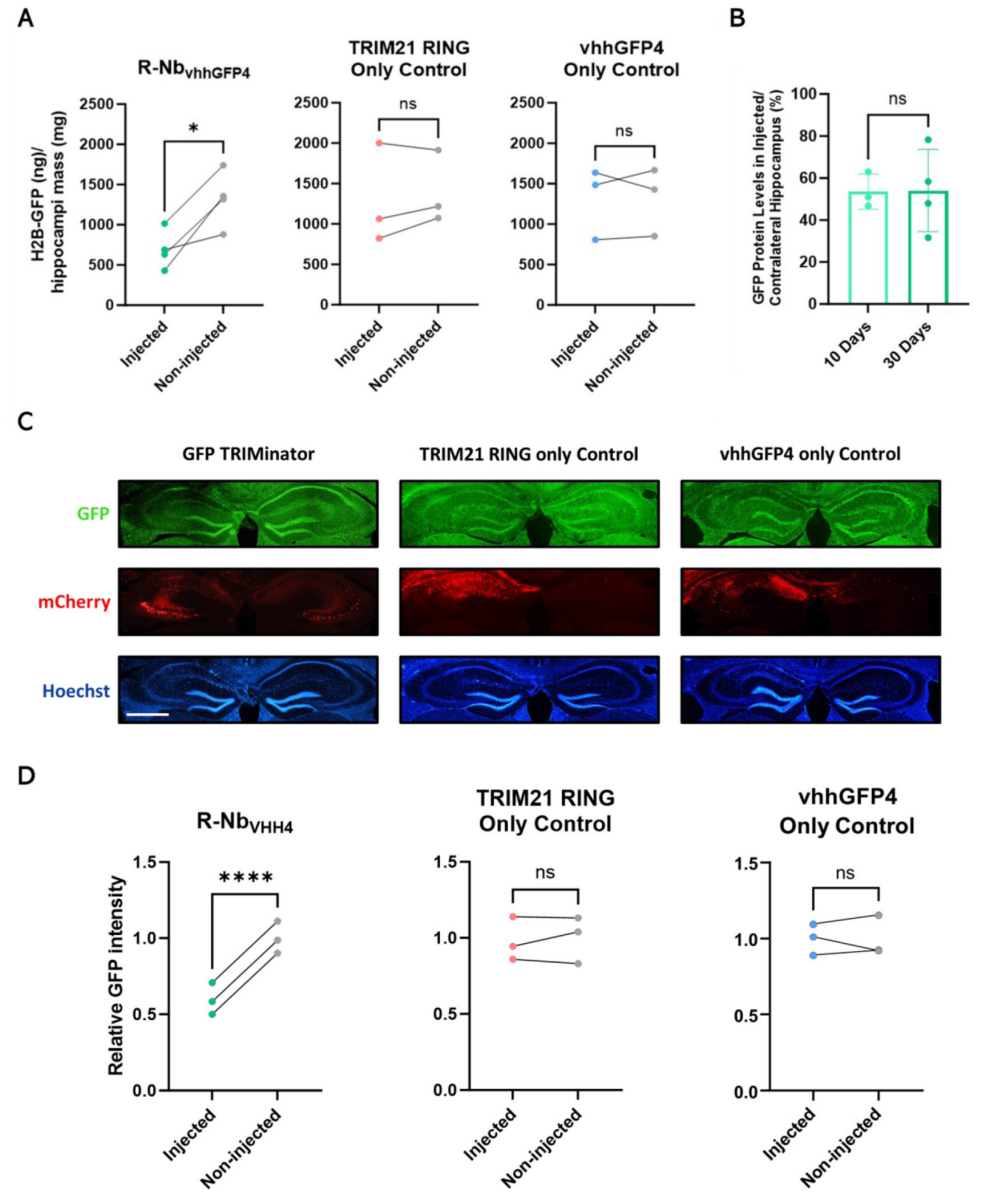
In vivo degradation of H2B-GFP in the mouse brain using R-Nb_vhhGFP4_. (**A**) H2B-GFP protein amounts in hippocampi of transgenic H2B-GFP mice unilaterally injected at two months of age with 2x10^9^ genome copies (GC) AAV1/2 (encoding R-Nb_vhhGFP4_, TRIM21 RING, or vhhGFP4) compared to non-injected contralateral hippocampi, quantified via GFP ELISA and normalized to hippocampus mass. (**B**) Comparison of H2B-GFP protein amounts in hippocampi injected with AAV1/2 R-Nb_vhhGFP4_ for 10 or 30 days, quantified via ELISA and presented as percentage H2B-GFP protein compared to non-injected contralateral hippocampi. (**C**) Representative fluorescent microscopy images of hippocampi injected with AAV1/2 encoding R-Nb_vhhGFP4_, TRIM21 RING, or vhhGFP4 30 days post-injection. Scale bar, 1mm. (**D**) GFP intensity in AAV1/2 injected vs non-injected hippocampi was quantified from fluorescent microscopy images and normalized against the average signal in non-injected hippocampi. (A) and (B) each point represents the average of two technical replicates from one mouse. n = 3 or 4 mice. (D) each point represents the average of 4 brain slices from one mouse. n = 3 mice. (A) and (D) paired *t* test, (B) un-paired *t* test. ns, not significant; *P < 0.05; ****P < 0.0001.

To investigate whether R-Nb constructs can degrade proteins in the brain, we targeted GFP-tagged histone 2B (H2B-GFP), an oligomeric substrate amenable to TRIM21 mediated degradation (14). AAV1/2 encoding a GFP-targeted degrader (R-Nb_vhhGFP4_) with a self-cleaving T2A-mCherry reporter was produced. TRIM21 RING and vhhGFP4 only were used as controls. Expression of all constructs was driven by a universal CAG promoter. AAV1/2 was injected unilaterally into the hippocampus of transgenic H2B-GFP mice at two-months of age. Thirty days later the hippocampi were removed, and homogenates prepared from injected and contralateral sides. GFP ELISA revealed that injection of AAV1/2 encoding R-Nb_vhhGFP4_ led to a reduction of H2B-GFP in injected vs contralateral hippocampi (Fig. 3A). To ascertain whether degradation can occur over a shorter time period, H2B-GFP expression was compared 10- and 30-days post AAV1/2 R-Nb_vhhGFP4_ injection. This revealed that H2B-GFP degradation in injected vs contralateral hippocampi already occurred 10 days post injection, with no difference compared to 30 days post injection, confirming the rapid nature of R-Nb mediated degradation (Fig. 3B). Fluorescence microscopy was also used to quantify H2B-GFP 30 days post AAV1/2 injection. Imaging confirmed that construct expression was largely confined to the injected hemisphere and that only AAV1/2 R-Nb_vhhGFP4_ was capable of reducing H2B-GFP expression (Fig. 3C and D). Thus, R-Nb constructs are amenable to AAV delivery and can be used to degrade oligomeric protein substrates in the CNS in vivo.

## Reduction of tau pathology in the aged mouse brain and primary neurons via R-Nb_F8-2_

**Fig. 4:**
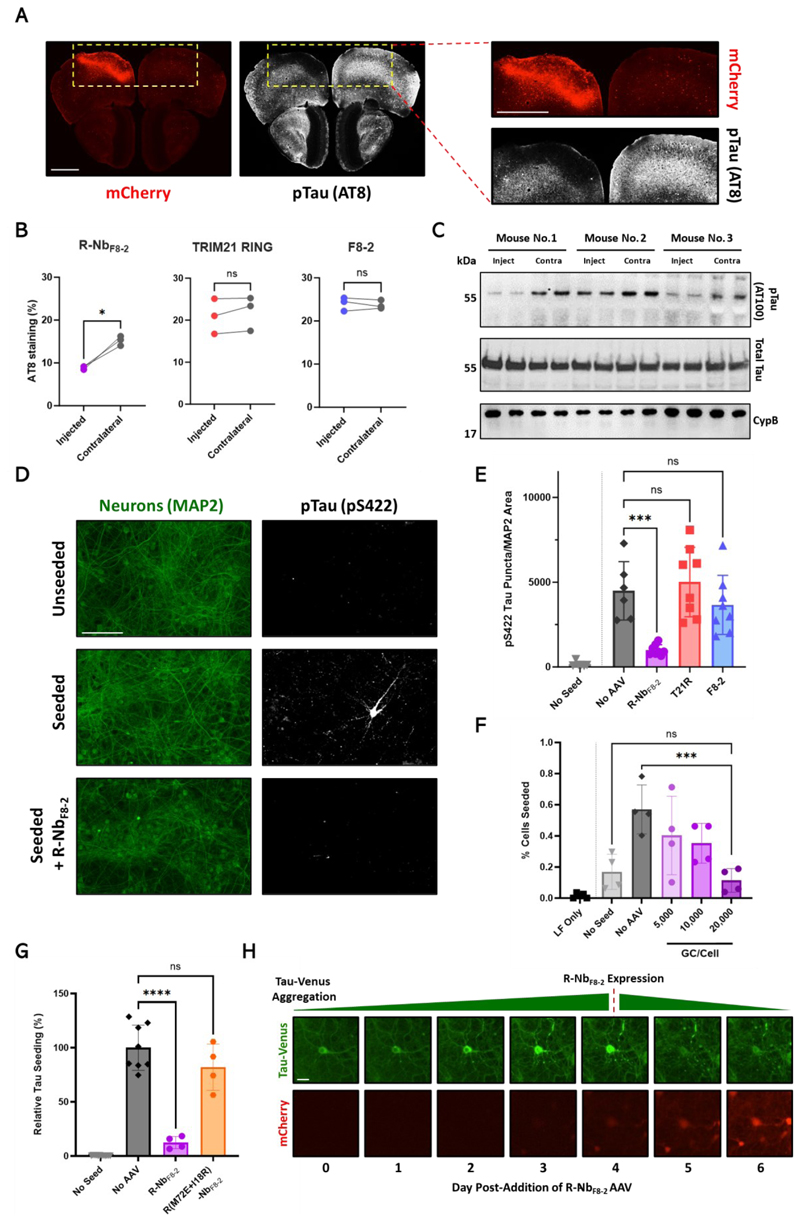
Reduction of tau pathology in the aged mouse brain and primary neurons via R-Nb_F8-2_. (**A**) Representative IF images 30 days post-stereotaxic injection of AAV1/2-CAG-R-Nb_F8-2_-T2A-mCherry into the left frontal cortex (two 2x10^9^ GC doses) of 5.5-month-old Tg2541 mice. pTau detected using AT8. Scale bars, 1mm. (**B**) Quantification of AT8 coverage in AAV1/2 injected vs contralateral frontal cortex hemispheres, 10 days post-injection. (**C**) Western blot of injected and contralateral cortical hemisphere lysates 10 days after injection with R-Nb_F8-2_ AAV1/2. Samples ran in duplicate and probed for pTau, total tau, and loading control with AT100, BR134 and CypB antibodies respectively. (**D**) Representative IF images of primary Tg2541 mouse neuron cultures, “seeded” via addition of 50 nM heparin-assembled P301S tau assemblies 7 days after pre-treatment with AAV1/2-hSyn-R-Nb_F8-2_-T2A-mCherry (20,000 GC/cell). pTau aggregates detected 7 days post-seeding via anti-pTau pS422 staining, with neurons identified via MAP2 staining. Scale bar, 100µm. (**E**) Quantification of pS422 tau puncta normalized against MAP2 coverage from IF images. Seeded neurons were pre-treated with AAV1/2 encoding R-Nb_F8-2_, TRIM21 RING (T21R) or F8-2 only. (**F**) Lysates from tau seeded neurons pre-treated with R-Nb_F8-2_ AAV1/2 (various GC doses) were introduced to TVS cells via Lipofectamine (LF). Tau-venus aggregation was quantified 72 hours later via fluorescent microscopy. (**G**) Relative amounts of pS422 tau puncta (normalized against MAP2 coverage) in tau seeded neurons pre-treated with catalytically dead or active R-Nb_F8-2_ AAV1/2 (20,000 GC/cell), compared to non-transduced neurons. (**H**) Primary C57BL/6 mouse neuron cultures were transduced with AAV1/2-hSyn-0N4R P301S tau-venus (20,000 GC/cell), seeded 7 days later with heparin assembled P301S tau assemblies, then transduced 3 days post-seeding with R-Nb_F8-2_ AAV1/2 (20,000 GC/cell), after which images were captured via live cell imaging. Scale bar, 20µm. Error bars represent mean ± SD. (B) n = 3 mice, 4 technical repeats per point. (E) and (G) n = 4 - 8 biological repeats, 2 technical repeats per point. (F) n = 4 biological repeats. (B) Paired *t* test. (E), (F) and (G) ANOVA with Tukey’s correction for multiple comparisons. ns, not significant; *P < 0.05; ***P < 0.001; ****P < 0.0001.

We next asked whether the tau-targeted R-Nb_F8-2_ degrader could be delivered via AAV to counter tau pathology in aged Tg2541 mice, which express human 0N4R P301S tau under the Thy1 promoter. AAV1/2 vectors bearing R-Nb_F8-2_ under a CAG promoter were injected unilaterally into the frontal cortex of 5.5-month-old mice, at which point their brains exhibit substantial AT8-positive tau pathology (Fig. S5A). Examination of brains 30 (Fig. 4A) or 10 (Fig. S5B) days later via immunofluorescence (IF) revealed that R-Nb_F8-2_ expression was largely restricted to the injected hemisphere, and that this was associated with a reduction in AT8 staining compared to the contralateral side. Expression of R-Nb_F8-2_, (but not TRIM21 RING or F8-2 only) reduced AT8 staining in the injected hemisphere (Fig. 4B). Frontal cortex hemispheres from mice treated with R-Nb_F8-2_ AAV1/2 were homogenized and probed for phosphorylated (pTau) (AT100, pThr212 & pSer214) and total tau protein, revealing that pTau amounts were reduced in injected hemispheres compared to contralateral sides; total tau remained unaffected (Fig. 4C).

We then assessed the ability of R-Nb_F8-2_ to prevent neuronal seeded tau aggregation using primary mouse neurons from neonatal Tg2541 mice. The addition of heparin assembled P301S tau fibrils to these cultures induced tau aggregation, visualized as pTau positive cell bodies and puncta via IF. Pre-treatment with AAV1/2 bearing R-Nb_F8-2_ under the neuron specific hSyn promoter reduced seeded aggregation (Fig. 4D and E). This reduction was observed in both neuronal cell bodies and dendritic compartments (Fig S5C and D). Conversely, pre-treatment with TRIM21 RING or F8-2 only AAV1/2 had no effect on seeded tau aggregation (Fig. 4E), despite similar expression as R-Nb_F8-2_ (Fig. S5E). Neither R-Nb_F8-2_ expression nor the AAV1/2 vector itself affected cell viability (Fig. S5F). To test whether R-Nb_F8-2_ expression reduced the formation of seed competent tau aggregates in this context, we tested lysates from tau-seeded neurons pre-treated with varying doses of R-Nb_F8-2_ AAV1/2 in TVS HEK cells. Results show that R-Nb_F8-2_ expression reduced the formation of seed competent tau in a dose-dependent manner (Fig 4F). To confirm that R-Nb_F8-2_ mediated neutralization of seeded tau aggregation is dependent on its E3 ligase catalytic activity, we introduced two RING-inactivating mutations (M72E and I18R) (26) into the R-Nb_F8-2_ construct. Pretreatment of neurons with AAV1/2 encoding this catalytically dead R-Nb_F8-2_ did not reduce seeded tau aggregation compared to non-transduced controls (Fig 4G), despite expression being similar to the active R-Nb_F8-2_ construct (Fig. S5G).

The ability of R-Nb_F8-2_ to reduce tau pathology in aged mice only days after AAV1/2 administration suggests that pre-existing neuronal tau aggregates are being degraded. To examine this possibility, primary mouse neurons expressing P301S tau-venus were treated with heparin assembled P301S tau aggregates and imaged longitudinally in situ. Seeded aggregates were detectable as bright venus puncta which, post-fixation, co-localized with AT8 staining (Fig. S5H). Addition of R-Nb_F8-2_ AAV1/2 caused large tau-venus aggregates to be rapidly cleared following R-Nb_F8-2_ expression (Fig. 4H and Supp. Video 2). These results demonstrate that R-Nb_F8-2_ expression can prevent and reverse tau aggregation in neurons, and can rapidly reduce tau pathology in an aged mouse model of tauopathy.

## Global CNS expression of R-Nb_F8-2_ reduces tau pathology in mice

**Fig. 5:**
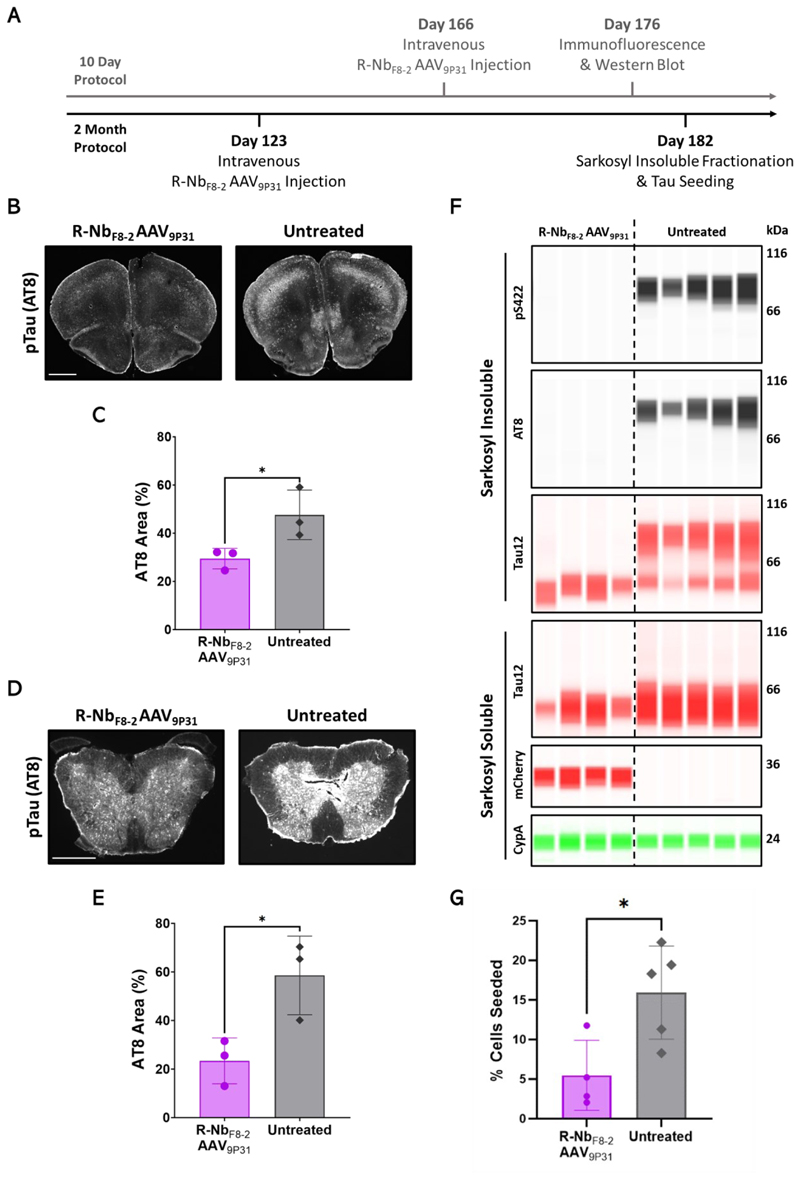
Global CNS expression of R-Nb_F8-2_ reduces tau pathology in mice. (**A**) Timeline of short (10 day) and long (2 month) experimental protocol using R-Nb_F8-2_ AAV_9P31_ in Tg2541 mice. In both scenarios mice were treated with a single dose of 1x10^11^ GC of R-Nb_F8-2_ AAV_9P31_ via tail vein injection. (**B**) Representative IF images of frontal cortices stained with AT8 from aged mice treated with or without R-Nb_F8-2_ AAV_9P31_ for 10 days. Scale bar, 1mm (**C**) Quantification of AT8 coverage from IF images in (B). (**D**) Representative IF images of spinal cords from the same mice, stained for AT8. Scale bar, 0.5mm (**E**) Quantification of AT8 staining in spinal cord sections (D). (**F**) Sarkosyl soluble and insoluble fractions were prepared from whole brains of aged mice treated with or without 1x10^11^ GC R-Nb_F8-2_ AAV_9P31_ for two months. Insoluble fractions were blotted for pTau (pS422 and AT8) and total tau (Tau12), with soluble fractions blotted for Tau12 and mCherry, with CypA as a loading control. Each lane represents an individual mouse. (**G**) Amounts of seeded tau aggregation in TVA cells treated with brain homogenates from aged mice treated with or without R-Nb_F8-2_ AAV_9P31_ for two months. Error bars represent mean ± SD. (C) and (E) each point represents the average of 4 brain slices from one mouse. n = 3 mice. (G) Each point represents the average percentage of TVS cells seeded (6 technical replicates) by homogenate from one mouse. n = 4 or 5 mice. Unpaired *t* test, *P < 0.05.

To be effective in clinical applications, broad transduction of the brain would likely be required to express R-Nb degraders in a majority of cells affected by tau pathology. In mice, this may be achieved through the use of engineered AAV capsids such as AAV_9P31_, a variant capable of transducing the brain following peripheral intravenous administration (27) (Fig. S6A). To determine the feasibility of this approach, R-Nb_F8-2_ was packaged into AAV_9P31_ under the hSyn promoter and delivered as a single tail vein dose to Tg2541 mice in either a short (10-day) or long (2-month) experimental protocol (Fig. 5A). In the 10-day protocol, R-Nb_F8-2_ AAV_9P31_ was administered to 5.5-month-old (Day 166) mice, with tau pathology assessed 10 days later via IF and western blot. AT8 staining of the frontal cortex revealed that R-Nb_F8-2_ AAV_9P31_ administration led to a decrease in pTau pathology compared to untreated mice (Fig. 5B and C). Tg2541 mice also develop severe spinal cord tau pathology by 5.5 months (28), which was reduced in R-Nb_F8-2_ AAV_9P31_ treated animals compared to controls (Fig. 5D and E). Immunoblotting of whole brain lysates (Fig. S6B) revealed decreased amounts of pTau in mice treated with R-Nb_F8-2_ AAV_9P31_ (Fig. S6C), whereas total tau remained unaffected (Fig. S6D). Similar results were obtained from spinal cord lysates (Fig. S6E, F and G).

In the two-month treatment protocol, four-month-old Tg2541 mice (Postnatal day 123) were injected with R-Nb_F8-2_ AAV_9P31_. At this age, brains from these mice exhibit moderate tau pathology (Fig. S6H). Two months later (Postnatal day 182), we prepared sarkosyl soluble and insoluble fractions from whole brain lysates. Insoluble tau phosphorylated at Ser396 (Fig. S6I), Ser422 and Ser202&Thr205 (AT8) was substantially reduced in R-Nb_F8-2_ AAV_9P31_ treated mice compared to untreated controls (Fig. 5F). Insoluble total tau running at the same weight as these phosphorylated species was also depleted in samples from treated mice. Soluble total tau was also reduced, albeit less strongly, in R-Nb_F8-2_ AAV_9P31_ treated mice; the cause of this effect remains to be determined. As previous, the delivery of brain lysates to TVS cells revealed that R-Nb_F8-2_ AAV_9P31_ treatment reduced seed competent tau species in the brain compared to untreated mice (Fig 5G). These results demonstrate that the R-Nb strategy can be used to reduce protein aggregates rapidly and durably at scale in the mammalian brain.

## Discussion

By fusing the E3 catalytic RING domain of TRIM21 to a target specific nanobody, we have engineered conditionally active degraders (R-Nb) that selectively degrade homotypic protein assemblies. R-Nb constructs using a tau specific nanobody were capable of rapidly clearing intracellular hyperphosphorylated and seed competent tau aggregates in various in vitro and in vivo models, whilst leaving native soluble tau largely unaffected. Degradation of a second substrate, histone 2B-GFP, demonstrates that the R-Nb approach can also achieve targeted protein degradation in the nucleus.

Fibrillar aggregates have long been considered challenging substrates for cellular degradation machinery. Previous studies have demonstrated that degradation by chaperones and the proteasome can produce an increased number of smaller aggregates, potentially mobilizing seed competent species (25, 29). We show here however that R-Nb degraders can successfully remove pre-formed insoluble tau aggregates, leading to a net decrease in seed-competent species. We speculate that the established role of TRIM21 in the destruction of cytosolic antibody-bound viruses in a time-sensitive manner likely renders it especially suited to be co-opted for this task.

The ability of R-Nb constructs to specifically target and deplete homotypic protein assemblies may aid in deconvoluting the contribution of assembled protein species towards neurodegenerative processes. Further studies assessing the phenotypic consequences of R-Nb mediated aggregate depletion are required for assessing aggregate-selective degraders as a therapeutic modality. This approach might, if successful, offer two key advantages over current strategies. First, unlike other interventions such as immunotherapy, these degraders are not reliant on intercepting extracellular tau assemblies, but are expressed within neurons to remove existing aggregates and prevent further aggregation. It would therefore be expected to operate irrespective of whether aggregation occurred via seeding or through cell-intrinsic mechanisms. Second, the monomer sparing quality of the approach offers a method by which the cellular functions of tau could be preserved, while pathological assemblies are removed. ASO studies suggest that tau depletion in the brain is well tolerated in phase I trials (4, 5). However, animal studies find phenotypes associated with total tau depletion, raising unknowns surrounding the effect of chronic depletion (30). Further studies exploring the functional consequences of aggregate-selective versus total tau depletion, alongside long-term toxicity profiling, will inform the feasibility of R-Nb constructs as a therapeutic modality against tau and other aggregating proteins in neurodegenerative diseases.

## Supplementary Material

Supplementary videos

Supplementary Materials

## Data Availability

All data are available in the manuscript or the supplementary material. Raw data has been uploaded to DRYAD (https://doi.org/10.5061/dryad.6m905qg89). Mass spectrometry raw files and data analysis files produced in Spectronaut have been uploaded to PRIDE (https://doi.org/10.1093/nar/gkab1038) under the accession number PXD052957.
