# Supplementary material for: Aggregate-selective removal of pathological tau via clustering-activated degraders
Source: Science. Author manuscript; Available in PMC 2024 Nov 19. (PMC7616837; doi:10.1126/science.adp5186)
Supplement: Supplementary Materials [file EMS200065-supplement-Supplementary_Materials.pdf]

Science
AAAS

Supplementary Materials for

Aggregate-selective removal of pathological tau via clustering-activated degraders

Jonathan Benn*[1†], Shi Cheng*[1†], Sophie Keeling[1], Annabel E Smith[1], Marina J Vaysburd[2], Dorothea Böken[1], Lauren VC Miller[2], Taxiarchis Katsinelos[1,2], Catarina Franco[2], Elian Dupré[3,4], Clément Danis[3,4,5], Isabelle Landrieu[3,4], Luc Buée[5], David Klenerman[1], Leo C James*[2,] William A McEwan*[1]

Corresponding authors: J.B: jb2310@cam.ac.uk. S.C: chengshi1013@163.com. W.M: wm305@cam.ac.uk. L.J: lcj@mrc-lmb.cam.ac.uk.

The PDF file includes:

Materials and Methods
Tables S1 to S3
Figs. S1 to S6

Other Supplementary Materials for this manuscript include the following:

Movies S1 to S2

Materials and Methods

Cloning

TRIM21 RING-Nanobody-T2A-mCherry (R-Nb) constructs were created using residues 1-85 of TRIM21 (encoding the RING E3 ligase domain) followed by a GGGGS linker connecting to the nanobody sequence. A C-terminal self-cleaving mCherry tag was added via inclusion of a T2A peptide (GSGEGRGSLLTCGDVEENPGP) prior to the mCherry sequence. R-Nb constructs were subcloned into pcDNA3.1+ (ThermoFisher, V79020) and pcDNA4.0/TO (ThermoFisher, V102020) for transient transfection and generation of doxycycline inducible cell lines respectively. R-Nb constructs were also subcloned into pAAV-CAG-GFP (Addgene plasmid #37825) and pAAV-hSYN-EGFP (Addgene plasmid #50465) vectors, replacing the GFP/EGFP transgene, for production of AAV particles carrying the R-Nb transgene.

Nanobody sequences used in R-Nb constructs (Table 1) were identified from the literature and synthesised as DNA fragments (GeneArt Strings, ThermoFisher) or generously provided by Elian Dupré and Luc Buée. Site directed mutagenesis (SDM) was used to make F8-2 variants.

Nanobody (Nb)	Target	Affinity (nM)	Reference
vhhGFP4	GFP	1	Kubala et al., 2010
MDQVQLVESGGALVQPGGSLRLSCAASGFPVNRYSMRWYRQAPGKEREWVAGMSSAGDRSSYEDSVKGRFTISRDD<br>ARNTVYLQMNSLKPEDTAVYYCNVNVGFEYWGQGTQVTVSS			
F8.2	Tau	1332	Dupré et al., 2019
MAEVQLQASGGGFVQPGGSLRLSCAASGGTSYWDGMGWFRQAPGKEREFVSAISGRGNIGTYYADSVKGRFTISRD<br>NSKNTVYLQMNSLRAEDTATYYCAAFRHEVHGSMRHEWEVIKYWGQGTQVTVSS			
F8.2 S54L T127A	Tau	683	Dupré et al., 2019, Fig. S2D
MAEVQLQASGGGFVQPGGSLRLSCAASGGTSYWDGMGWFRQAPGKEREFVSAI**L**GRGNIGTYYADSVKGRFTISRD<br>NSKNTVYLQMNSLRAEDTATYYCAAFRHEVHGSMRHEWEVIKYWGQGTQV**A**VSS			
F8.2 R111C	Tau	211	Dupré et al., 2019
MAEVQLQASGGGFVQPGGSLRLSCAASGGTSYWDGMGWFRQAPGKEREFVSAISGRGNIGTYYADSVKGRFTISRD<br>NSKNTVYLQMNSLRAEDTATYYCAAFRHEVHGSM**C**HEWEVIKYWGQGTQVTVSS			
H3-2	Tau	32	Fig. S2 A-C
MAEVQLQASGGGFVQPGGSLRLSCAASGYTSGDEIMGWFRQAPGKEREFVSAISWQSGTSTYYADSVKGRFTISRD<br>NSKNTVYLQMNSLRAEDTATYYCAPMTLAETYYEWLISGYWGQGTQVTVSS			
Nb139	p53	N/A (for Tau-Venus)	Bethuyne et al., 2014
AQVQLQESGGGLVQAGGSLRLSCAASERTFSTYAMGWFRQAPGREREFLAQINWSGTTTYYAESVKDRTTISRDNA<br>KNTVYLEMNNLNADDTGIYFCAAHPQRGWGSTLGWTYWGQGTQVTVSS			

Table S1. Nanobodies (Nb) used in R-Nb constructs.

Nanobody name, target, affinity and reference are given along with protein primary sequence. Bold residues indicate mutations in F8-2 variants.

Cell line creation and culture

HEK293 T-REx cells (ThermoFisher, R71007) were cultured in complete DMEM supplemented with 10% vol/vol foetal bovine serum (FBS), 100 U/ml penicillin and 100 μg/ml streptomycin at 37°C with 5% CO2 in a humid atmosphere. TVS cells expressing soluble human 0N4R P301S tau with a C-terminal venus fluorophore (*8*) were transfected with sarkosyl insoluble tau assemblies isolated from 6 month old Tg2541 P301S tau transgenic mice and clonally selected to isolate a line in which tau-venus was constitutively aggregated (TVA cells).

TVS and TVA cells expressing DOX inducible R-Nb constructs were created by stable transfection with R-Nb pcDNA4/TO plasmids using Lipofectamine 3000 (ThermoFisher, L3000015), followed by selection in media containing 50μg/ml Zeocin (AlfaAesar, J67140). Construct expression was induced via the addition of 1μg/ml DOX (MP Biomedicals), 195044) in the media. Small molecule inhibitors of the UPS/VCP/Autophagy (solubilised in DMSO) were added at the following concentrations in the media alongside 1μg/ml DOX: TAK-243 (100nM); HOIPIN-8 (25μM); MG132 (125nM); NMS-873 (5μM); Bafilomycin A1 (400nM).

Recombinant tau protein production

The expression and purification of recombinant human 6xHis-0N4R tau bearing the P301S mutation from E. coli BL-21 (DE3, Agilent Technologies) was performed as described previously (*31, 32*) with small modifications. Bacterial pellets were collected through centrifugation (3300 g, 4 °C, 10 min) and then resuspended in 10 ml/L of culture with buffer A (25 mM HEPES, pH 7.4, 300 mM NaCl, 20 mM Imidazole, 1 mM benzamidine, 1 mM PMSF, 14 mM β-mercaptoethanol, 1% NP-40, 1 x complete EDTA-free protease inhibitors). The resuspended bacteria were lysed on ice using a probe sonicator and boiled for 10 min at 95 °C which denatures the majority of proteins, but not tau. Denatured proteins were pelleted by ultracentrifugation at 100,000 g, 4 °C for 50 min. The clarified supernatant containing 6xHistagged monomeric tau P301S was then passed through a HisTrap FF column according to manufacturer instructions (GE Healthcare). Eluted fractions were assessed through SDS-PAGE and total protein staining with Coomassie InstantBlue. Fractions of interest were concentrated using 10 kDa cut-off Amicon Ultra-15 concentrators (Merck Millipore) before loading on a HiLoad 16/600 Superdex 200 (Cytiva) size exclusion chromatography column. To remove the 6xHis tag, tau was subjected to TEV protease following manufacturer's instructions (Sigma-Aldrich, T4455). After incubation, the pooled fractions were loaded onto a second HisTrap HP column to remove protease and the successfully cleaved tau collected. Cleaved tau was concentrated using a 10 kDa cut-off Amicon Ultra-15 concentrator (Merck Millipore) before loading onto a HiLoad 16/600 Superdex 200 (Cytiva) size exclusion chromatography column. All purification was performed on an ÄKTA Pure system (Cytiva).

Purified tau was concentrated to at least 3 mg/mL using a 10 kDa cut off AmiconUltra-15 concentrator (Merck Millipore) and snapfrozen in liquid nitrogen for storage at -80 °C in PBS containing 1 mM DTT. Levels of endotoxin were measured using a limulus amebocyte lysate kit and found to be below 0.01 EU/ml at tau concentrations used in experiments.

In vitro aggregation of recombinant tau protein

Tau monomer was aggregated as described previously (*31*). Briefly, 60 μM tau monomer was incubated with 20 μM heparin, 2 mM DTT and 1X protease inhibitors in PBS for 24–72 h at 37°C shaking at 250 RPM. Thioflavin T (ThT, Thermo Fisher, T3516) was used to quantify Tau aggregation. Tau assemblies were then diluted in PBS to 20 μM monomer equivalent, snap-frozen in liquid nitrogen and stored at -80 °C.

Quantification of tau-venus aggregation in cell lines via fluorescent microscopy

20,000 TVA cells per well of a 96-well plate were reverse transfected with pcDNA3.1+ plasmids encoding R-Nb constructs. 24 hours later, live cells were washed 2x with PBS and incubated with 2μg/ml Hoechst (Invitrogen, H3570) in PBS for 30 minutes at 37°C. Images were then acquired on an Eclipse Ti2 Microscope (Nikon) using a 10x objective with the percentage of cells containing tau-venus aggregates, and mean cell mCherry fluorescence, being quantified using NIS-elements software (Nikon). Quantitative live cell imaging of TVA cells expressing DOX inducible constructs was performed similarly, with cells being plated the day before DOX addition and then imaged every 30 minutes post-DOX addition for 20 hours. Videos and representative images were acquired from cells cultured on glass coverslips and imaged on a confocal microscope (STELLARIS8, Leica), with images being acquired every 20 minutes from 4 to 15 hours post DOX addition.

Seeded tau aggregation in TVS cells induced by heparin assembled tau assemblies was performed largely as described previously (*11*). 15,000 TVS cells were plated per well of a 96-well plate in 50μL OptiMEM (ThermoFisher, 31985070) and treated with varying concentrations of tau assemblies diluted in a further 50μL OptiMEM containing 0.5μL Lipofectamine 2000 (ThermoFisher, 11668019). After 1 hour, 100μL complete DMEM was added to each well to stop the transfection process. 72 hours later, live cells were washed 2x with PBS and incubated with 2μg/ml Hoechst (Invitrogen, H3570) in PBS for 30 minutes at 37°C. Images were then acquired on an Eclipse Ti2 Microscope using a 10x objective with the percentage of cells containing tau-venus aggregates being quantified using NIS-elements software.

A similar protocol was used to quantify seeded tau aggregation induced by various homogenate samples. HEK293 and neuronal cultures were lysed via the addition of ice-cold PBS containing 1x Halt™ protease and phosphatase inhibitor cocktail (ThermoFisher, 78440) and 5 cycles of freeze-thaw between -80°C and 37°C, after which membranes were pelleted and supernatants collected. The preparation of mouse brain homogenates is described later (*Preparation of homogenates and sarkosyl soluble and insoluble fractions from HEK293T cells and mouse brains*). The total protein concentration in all homogenates was quantified via BCA and adjusted with PBS to 20ng/μL for samples from HEK293 and neuronal cultures, and to 100ng/μL for mouse brain

homogenates. Samples were then transfected into TVS cells following the same protocol as transfection of heparin assembled tau assemblies.

Flow Cytometry

100,000 TVS cells were plated per well of a 24-well plate and reverse transfected with pcDNA3.1+ plasmids encoding R-Nb constructs. 24 hours later, cells were collected into a round bottom 96-well plate, pelleted, and then resuspended in 150µL PBS. This plate was loaded into a CytoFLEX (Beckman Coulter) flow cytometer where 10,000 events per condition were recorded. Cells were gated to be live singlets and have equal mean mCherry expression, with analysis and gating being performed on CytExpert software (Beckman Coulter).

Preparation of homogenates and sarkosyl soluble and insoluble fractions from HEK293T cells and mouse brains

HEK293 homogenates were prepared via addition of M-PER™ Mammalian Cell Lysis Buffer (ThermoFisher, 78501) followed by one freeze-thaw cycle between -80°C and room temperature. Lysates were cleared by centrifugation and supernatants collected for analysis.

Mouse brains were homogenised in ice-cold H-Buffer (10 mM Tris pH 7.4, 1 mM EGTA, 0.8 M NaCl, 10% w/v sucrose, 1x protease and phosphatase inhibitors) in a 6:1 volume (µL):brain mass (mg) ratio using the VelociRuptor V2 Microtube Homogeniser (Scientific Laboratory Supplies). Homogenisation was completed with a KIMBLE® pestle homogeniser (DWK Life Sciences) prior to samples being lysed by sonication and clarified by centrifugation.

Sarkosyl soluble and insoluble fractions were extracted from HEK293 cells (TVA), and mouse brain homogenates as previously described (*33*). TVA cell homogenates were prepared by resuspending cells (from one 10cm dish) in 1ml H-Buffer (plus 1% Sarkosyl), then lysing cells via sonication. Mouse brain homogenates, prepared as described above, had sarkosyl added up to a final concentration of 1%. To isolate sarkosyl soluble and insoluble fractions from these homogenates (TVA or mouse brain), samples were incubated for 1 hour at 37°C and then centrifuged at 100,000x g for 1 hour at 4°C. Sarkosyl soluble fractions were collected and the resulting sarkosyl insoluble pellet was resuspended in 15µL tris-buffered saline (TBS) for TVA samples, and in 0.2ml/gram of original brain tissue for mouse brain derived samples, after which all samples were sonicated before storage at -80°C.

Capillary-based immunoblot

Proteins of interest in homogenates or sarkosyl soluble/insoluble fractions from HEK293 cells or mouse brain samples were detected using the automated Jess capillary-based immunoblot platform (Bio-Techne). Samples were mixed with Jess 5x fluorescent master mix and boiled at 100°C for 5 minutes, with 4µL of sample then being ran on the Jess system as per the manufacturer's instructions. Primary and Secondary antibodies used are described in Table 2. Band intensities were quantified using Simple Western software (Bio-Techne).

Antibody Target	Antibody Species	Antibody Dilution	Supplier & Catalogue No.
Total Tau (Tau12)	Mouse	1:100	Sigma-Aldrich, MAB2241
Total Tau (DAKO)	Rabbit	1:100	Dako, A0024
Total Tau (HT7)	Mouse	1:100	Invitrogen, MN1000
pTau (AT8)	Mouse	1:25	Invitrogen, MN1020
pS422	Rabbit	1:25	Abcam, ab79415
pS396	Rabbit	1:15	Invitrogen, 44-752G
mCherry	Rabbit	1:50	Proteintec, 26765-1-AP
GAPDH	Rabbit	1:100	Bio-Techne, 2275-PC-100
Actin	Mouse	1:50	Bio-Techne, MAB8929
CypA	Goat	1:40	Bio-Techne, AF3589
2A Peptide	Mouse	1:25	Merck, MABS2005
Anti-Mouse IgG (647 Secondary)	Donkey	1:20	Invitrogen, A32787
Anti-Mouse IgG (HRP Secondary)	Donkey	1:20	Invitrogen, A16017
Anti-Rabbit IgG (800 Secondary)	Donkey	1:20	Invitrogen, A32808
Anti-Rabbit IgG (647 Secondary)	Donkey	1:20	Invitrogen, A32795
Anti-Rabbit IgG (HRP Secondary)	Donkey	1:20	Jackson, 711-035-152
Anti-Goat IgG (800 Secondary)	Donkey	1:20	Rockland, 605-745-125

Table S2. List of primary and secondary antibodies used with automated Jess capillary-based immunoassay system.

Western Blot

Proteins of interest in HEK293 cell or mouse brain homogenates were also detected via western blot. 4x NuPAGE LDS sample buffer (ThermoFisher, NP0007) with 2mM β-mercaptoethanol was added to samples before boiling for 5 mins, after which samples were subjected to SDS-PAGE using NuPAGE Bis-Tris 4-12% gels (ThermoFisher, NP0324BOX) and transferred to 0.2µm PVDF membrane using the Bio-Rad Transblot Turbo Transfer System. Membranes were blocked in 5% milk with 0.2x fish gelatine in TBS-T (0.1% Tween-20 in TBS) for 1 hour at room temperature before incubation with primary antibodies (Table 3). Membranes were incubated in primary antibody overnight at 4 ºC and following repeated washes with TBS-T, were incubated with secondary antibodies (Table 3) for 1 hour at room temperature. Membranes then washed with TBS-T and incubated with HRP substrate where appropriate (Millipore, WBKLS0500), before imaging on a ChemiDoc system (BioRad). Band intensities were quantified using ImageJ Software.

Antibody Target	Antibody Species	Antibody Dilution	Supplier & Catalogue No.
pTau (AT100)	Mouse	1:1000	Invitrogen, MN1060
Total Tau (BR134)	Rabbit	1:1000	Goedert et al., 1989
CypB	Mouse	1:5000	Santa Cruz, K2E2
Ubiquitin	Mouse	1:2000	ThermoFisher, MA1-10035
LC3B	Rabbit	1:1000	Merck, L7543
GAPDH	Mouse	1:5000	Abcam, Ab8245
Mouse IgG (HRP Secondary)	Goat	1:8000	Invitrogen, A16011
Rabbit IgG (HRP Secondary)	Goat	1:8000	Invitrogen, A16023
Mouse IgG (Dylight 800 Secondary)	Goat	1:5000	ThermoFisher, SA5-10176
Rabbit IgG (Dylight 800 Secondary	Goat	1:5000	ThermoFisher, SA5-10036

Table S3. List of primary and secondary antibodies used for western blot.

Immuno-gold electron microscopy

Sarkosyl insoluble species from TVA cells were applied on glow-discharged 400 mesh formvar/carbon film-coated copper grids (EM Sciences, CF400-Cu) for 45 secs. Immunogold labelling was performed as described previously (*34*). Briefly, grids with deposited samples were blocked at room temperature for 10 min with PBS + 0.1% fish gelatine (G7041, Merck) and then incubated with anti-tau (BR134) (*31*) or anti-GFP (Ab6556, Abcam) antibodies diluted in blocking buffer (1:50) for 1 hour at room temperature. Grids were subsequently washed with blocking buffer and incubated with 10nm gold-conjugated anti-rabbit IgG secondary antibody (G7402, Merck) for 1 h at room temperature diluted 1:20 in blocking buffer. Grids were finally washed with water, stained with 2% uranyl-acetate for 45 secs and air-dried for at least 30 min before imaging. Images were acquired at 4,400× and 6,500× with a defocus value of -1.4 µm with Gatan Orius SC200B detector using a Tecnai G2 Spirit at 120 kV.

Single molecule pull-down (SiMPull) super-resolution microscopy

Single molecule pull-down (SiMPull) super-resolution microscopy was performed as previously described (*21*). Briefly, passivated PEGylated glass coverslips were coated with 10nM biotinylated Ht7 (Invitrogen, MN1000B) capture antibody diluted in blocking solution (1mg/mL BSA in PBS) for 10 minutes. This was followed by washing in PBST (0.05% Tween-20 in PBS: 50mM tris base, 150 mM NaCl, pH 7.4) and 10 minutes incubation in blocking solution. After another wash in PBST, 10µL of sample (1:50 dilution in PBS) was added and left to incubate for 1 hour at room temperature, followed by washing in TBST. Ht7 labelled with a covalently coupled docking strand (DBCO TEG-AAACCACCACCACCACCACCACCACCACCACCACCA) was used for detection at 3nM in blocking solution, being incubated on the slide for 15 minutes, followed by a

final wash in PBS. 3μL imaging strands (1 nM in PBS, TATGTAGATC-AminoC7 - Atto655) were then added before the gasket was sealed with another clean coverslip.

Imaging was done on a home-built total internal reflection fluorescence (TIRF) microscope, consisting of an inverted Ti-2 Eclipse microscope body (Nikon) fitted with a 1.49 N.A., 60x TIRF objective (Apo TIRF, Nikon) and a perfect focus system. Images were acquired using a 638 nm laser (Cobolt 06-MLD-638, HÜBNER). Images were collected in a grid using an automated script (Micro-Manager) to avoid any bias in the selection of FOVs. Images were acquired for 7000 frames of 100 ms exposure. Super-resolution images were reconstructed using the Picasso package. Localizations were identified and fit using IFComDet then corrected for microscope drift using the inbuilt implementation of redundant cross-correlation. Localizations were then filtered for precision <30 nm and then clustered using DBSCAN as provided by the scitkit-learn package with permissive parameters (radius of 0.25 and minimum density of 5). The skeletonized length of each aggregate was measured whereby the length of each aggregate is reported as the summed branch distance. Finally, super-resolved images were rendered using the inbuilt Picasso functionality.

Mass Spectrometry

For mass spectrometry analysis, 50ug of each cell lysate sample was diluted to 1μg/μL using 25mM AMBIC. Cysteines were reduced by adding DTT to a final concentration of 4mM and heating the samples to 60°C for a total of 10 minutes. To prevent cysteine re-oxidation iodoacetamide was added as an alkylating reagent to a final concentration of 14mM and incubation proceeded for 45 minutes at room temperature in the dark. Digestion was carried out semi-automatically on a Kingfisher Apex using the Protein Aggregation Capture method adapted from (*35*). Briefly, reduced and alkylated samples were transferred to a 96 well plate and precipitated by adding acetonitrile to a final concentration of 70% (v/v). Washed MagResyn Hydroxyl microparticles from Resyn Biosciences were immediately to the samples at a ratio of 1:4 (protein:bead) to promote protein precipitation and on-bead aggregation. Three subsequent washes of the beads with the aggregated proteins was performed with 100% acetonitrile and followed by two additional washes with 70% ethanol. In-bead digestion was performed on a first stage on the Kingfisher Apex by adding 1μg of trypsin to 100uL of 25mM AMBIC containing 0.2% (v/v) RapiGest detergent (Waters) and incubating for 1h at 47°C. Overnight digestion was then carried out at 37°C on an Eppendorf ThermoMixer C for an additional 16h. Magnetic beads were removed and peptides were acidified with the addition of trifluoroacetic acid to a final concentration of 0.5% (v/v). The acidified tryptic digest was then centrifuged at 13,000 x *g* for 15 minutes to remove RapiGest degradation by-products and the supernatant subjected to LC-MS/MS analysis.

LC-MS/MS was performed on an Vanquish Neo UHPLC (ThermoFisher Scientific, San Jose, USA) hyphenated to an Orbitrap Eclipse mass spectrometer (ThermoFisher Scientific, San Jose, USA). Peptides were trapped on a C18 Acclaim PepMap 100 (5 μm, 300 μm x 5mm) trap column (ThermoFisher Scientific, San Jose, USA) and separated on a C18 Aurora Ultimate TS (25cm x 75 μm) column (IonOpticks, Australia) over a gradient of solvent B [80 % (v/v) acetonitrile, 0.1% formic acid] from 3% to 25% B over 135 minutes followed by a 25% to 45 %B over 45minutes. MS1 full scans were acquired in the Orbitrap at a resolution of 120,000 (AGC target of 4e5 ions

with a maximum injection time of 50ms) and followed by MS2 in a data-independent acquisition setting composed of 41 staggered variable width window covering 400-900 m/z. MS2 DIA scans were acquired in the Orbitrap at a resolution of 30,000 with a maximum injection time of 54ms and an HCD collision energy of 30%.

Raw data were imported and processed in Spectronaut 18.0 (Biognosys) using stringent criteria as set out in (*36*). Raw files were searched against *Homo sapiens* protein sequences downloaded from UniProt (UP000005640_9606) with the overexpressed proteins appended to the protein database (TauP301S-Venus; R-NbF8-2 and mCherry). Differential abundance testing was performed using unpaired t-test with group-wise testing correction.

AAV preparation

Chimeric particles of adeno-associated virus produced with capsids of types 1 and 2 (AAV1/2) were prepared as previously describe (*37*). 7µg pAAV-hSyn/CAG-Transgene, 3.5µg pAAV2/1 Rep-Cap, 3.5µg pAAV2/2 Rep-Cap and 20µg pAdDeltaF6 helper plasmid per plate were co-transfected in 10×15 cm cell culture dishes of 60% confluent AAVpro® HEK293T cells (Takara, 632273) via polyethylenimine (PEI). After 60 hours, medium was collected and cells resuspended in 10 mL total AAV lysis buffer (20mM Tris pH 8.0, 1mM $MgCl_2$, 150mM NaCl). NaCl (0.93 g) and 10mL of 40% polyethylene glycol 8000 (PEG) was added per 40mL media and incubated on ice overnight, after which AAV was precipitated via centrifugation and resuspended in 4 mL total AAV lysis buffer and pooled with the cell pellet. Combined resuspended pellets were incubated with 1mM $MgCl_2$ and 100U benzonase for 15 mins at 37°C and then freeze-thawed 3 times before a final benzonase digestion with an additional 100U. This suspension was then centrifuged, with AAV containing supernatants being collected and then subjected to iodixanol (Optiprep) (Sigma-Aldrich, D1556) gradient ultracentrifugation. Iodixanol was layered into a 38.5mL ultracentrifuge tube (Beckman, 344326) with the following solutions: 6mL of 17% (5mL 10x PBS, 0.05mL 1M $MgCl_2$, 0.125mL 1M KCl, 10mL 5M NaCl, 12.5mL Optiprep, H_2O to 50mL), 6mL of 25% (5mL 10x PBS, 0.05mL 1M $MgCl_2$, 0.125mL 1M KCl, 20mL Optiprep, 0.1mL of 0.5% phenol red, H_2O to 50mL), 5mL of 40% (5mL 10x PBS, 0.05mL 1M $MgCl_2$, 0.125mL 1M KCl, 33.3mL Optiprep and H_2O to 50mL) and 6mL of 60% (0.05mL 1M $MgCl_2$, 0.125mL 1M KCl, 50mL Optiprep, 0.025mL 0.5% phenol red). AAV containing supernatants were then layered on top of the 17% layer and ultracentrifuged (68,000 RPM, 17°C, 70 mins) in a Type 70Ti rotor (Beckman, 337922). The 40% iodixanol fraction was isolated and concentrated to ~100 µL in PBS, with single use aliquots being frozen at −80°C. AAV titers were determined via SYBR® Green qPCR (ThermoFisher, 4344463) according to the manufacturers protocol using forward (GGAACCCCTAGTGATGGAGTT) and reverse (CGGCCTCAGTGAGCGA) primers for the AAV ITR regions. AAV purity was determined via SDS-PAGE followed by Coomassie staining. AAV_{9P31} was prepared following the same protocol, but replacing the AAV2/1 and AAV2/2 Rep-Cap plasmids with an AAV9 Rep-Cap with a WPTSYDA heptapeptide inserted between residues 558 and 589 of the VP1 gene via site directed mutagenesis (*27*).

Mice and AAV injections

All animal work was licensed under the UK Animals (Scientific Procedures) Act 1986 and was approved by the Medical Research Council Animal Welfare and Ethical Review Body. H2B-GFP transgenic mice (CAG:H2B-EGFP) were obtained from Jackson Laboratory (MGI:3686857). Tg2541 transgenic mice (MGI:3778191), which express human 0N4R P301S tau under the control of a Thy1 promoter (*19*), were obtained from Dr Michel Goedert, MRC Laboratory of Molecular Biology, UK. A spread of litters and sexes were used for all primary culture and in vivo experiments. Power analysis for in vivo group sizes was performed using pilot data with alpha = 0.05; beta = 0.8.

Stereotaxic injection of $2x10^9$ genome copies (GC) AAV1/2 into the left hippocampus of 2-month-old H2B-GFP transgenic mice was performed using coordinates: AP: -1.60, ML: +1.36, DV: −1.2mm. Two injections of $2x10^9$ GC were administered to the left frontal cortex of 5.5-month-old Tg2541 tau transgenic mice at the coordinates: AP: +2.96, ML: +0.75, DV: -0.75 mm; and AP: +2.46, ML: +1.64, DV: -1.13 mm. $1x10^{11}$ GC AAV-9P31 was injected intravenously through the tail vein of 4- or 5.5-month-old Tg2541 tau transgenic mice. All injections were performed under anesthetic using isoflurane inhalation (induction = 2% isoflurane 1L/minute O2, maintain = 1% isoflurane 1L/minute O2).

For performing histological analysis, mice were culled by exsanguination and perfused with pre-cooled 4% PFA. Brain and spinal cord samples were collected in PFA overnight, dehydrated in 30% sucrose in PBS (w/v) and then stored at -80°C until slicing into 30µm sections on a cryostat. For immunoblot analysis, mice were culled by exsanguination with samples were collected in pre-cooled PBS and then stored at -80°C.

GFP ELISA

Injected and non-injected H2B-GFP mouse hippocampi were isolated and separately homogenised as described above. H2B-GFP protein levels in these homogenates were quantified using a GFP ELISA kit (Abcam, ab171581) as per the manufacturer's instructions.

Fluorescent microscopy and immunofluorescence of mouse brain sections

Brain/Spinal cord slices were mounted onto Superfrost Plus (ThermoFisher, 10149870) glass slides. For IF staining, mounted slices were blocked and permeabilised for one hour using PBS containing 10% goat serum and 0.3% Triton X-100, followed by incubation with primary antibodies in antibody diluent (PBS containing 1% goat serum and 0.003% Triton X-100) overnight at 4°C on a shaking incubator in the dark. Biotin conjugated mouse AT8 (Invitrogen, MN1020B) was used at 1:1200, with the rabbit Dako anti-total tau antibody (Dako, A0024) being used at 1:1000. Following this overnight incubation, primary antibodies were removed, and slides washed in PBS. Streptavidin conjugated Alexa Fluor 647 (Invitrogen, S32357) (1:500) and goat anti-rabbit Alexa Fluor 647 (Invitrogen, A21245) were then incubated with slides in antibody diluent for 2 hours at room temperature, after which secondary antibodies were removed and slides washed again in PBS. Hoechst 33342 (1µg/ml in PBS) was then added at for 5-10 minutes to stain

nuclei, followed by a final wash in PBS. Coverslips were then added to slides, and images acquired the next day using an Eclipse Ti2 microscope, with AT8/GFP coverage being quantified in ImageJ.

Primary mouse neuron cultures

Brains were removed from the heads of P0 or P1 mice and pooled cortex and hippocampal neuronal cultures were prepared as previously described (*38*, *39*). Hippocampi and cortices were dissected in ice cold Hibernate-A (Gibco, A1247501) and the meninges removed. Tissues were pooled in a 15mL conical tube and washed twice with room temperature Hibernate-A before being incubated with a final concentration of 0.25% trypsin (Gibco, 15090-046), at 37°C for 20 minutes. During this period, a cotton-plugged glass Pasteur pipette (Merck Life Science, S6143) was fire-polished. Following trypsinisation, 500µL 1% (w/v) DNAse I (Sigma-Aldrich, DN25) was added to the tissue and incubated at room temperature for 5 minutes. The tissue was washed twice with 37°C Hibernate-A, followed by two washes with 37°C neuron plating medium (PM) containing Neurobasal Plus (Gibco, A3582901), 1 mM GlutaMAX (Gibco, 15050061), 1% penicillin-streptomycin (Invitrogen, 15140122), 10 % horse serum (Invitrogen, 26050070), and 1x B-27 Plus supplement (Gibco, A352801). After washing, 2.5 ml PM was added to the tissue, and the tissue was triturated using the glass pipette in a 60 mm dish. A further 8 mL of PM was added to the dish, and the cell suspension passed through a 70 µm cell strainer. Live cells were counted via trypan blue staining using the Countess II automated cell counter (Invitrogen). For tau seeding experiments, 30,000 cells/well were seeded into black 96-well plates (Greiner Bio-One, 655090), coated with poly-L-lysine (RnD Systems, 3438-100-01). After 4 h, all media was removed and replaced with maintenance media (MM) (PM without serum). All primary cultures were maintained in a humidified tissue culture incubator at 37 °C with 5 % CO2.

Quantification of seeded tau aggregation in primary mouse neurons

Seeded tau aggregation was induced in primary mouse neurons via the addition of 50nM recombinantly produced 0N4R P301S-tau assemblies in maintenance media and quantified via IF. Media was removed from neurons followed by two washes with ice cold PBS, after which neurons were fixed and permeabilised via the addition of 100µL/well ice cold methanol for 3 minutes on ice. Following this incubation, 100µL PBS was added to dilute the methanol, and then 100µL of this mixture was removed. This dilution step was repeated a total of three times, after which all liquid was removed from the well. Fixed neurons were then washed with PBS and blocked with 2% BSA in PBS (Neuron IF block) for 30 minutes at room temperature.

Primary antibody solutions were made up in neuron IF block at the following concentrations: Rabbit anti-phospho-tau serine 422 (pS422) (Abcam, ab79415) 1:1000; Chicken anti-MAP2 (Abcam, ab5392) 1:5000; Mouse anti-phospho-tau serine 202 and threonine 205 (AT8) (Invitrogen, MN1020) 1:1000. Neurons were incubated with primary antibody overnight at 4°C. The following day, primary antibody was aspirated, and cells washed with PBS. Secondary antibody solutions were made up in neuron IF block at the following concentrations: goat anti-rabbit Alexa Fluor 647 (Invitrogen, A21245) 1:500; goat anti-chicken Alexa Fluor 488 (Invitrogen, A11039) 1:500; goat anti-mouse Alexa Fluor 647 (Invitrogen, A32728) 1:500; goat anti-chicken Alexa Fluor 568 (Invitrogen, A11041) 1:500. Neurons were incubated with secondary antibody

for 1 hour at room temperature in the dark, after which antibody was removed and neurons were washed with PBS. Finally, 2μg/ml Hoechst 33342 (diluted in PBS) was added to neurons for 10 minutes at room temperature (in the dark), followed by a final wash step in PBS. Cells were left in PBS for fluorescence imaging using an Eclipse Ti2 Microscope. NIS-Elements software (Nikon) was used to quantify pS422 puncta (puncta detection), pS422 cell bodies (thresholding) and MAP2 coverage in each image. Tau seeded primary neurons treated with AAV1/2-hSyn-0N4R P301S tau-venus and AAV1/2-hSyn-R-Nb$_{F8\text{-}2}$-T2A-mCherry were live imaged in an IncuCyte® S3 Live-Cell Analysis System.

HEK Cell Immunofluorescence (IF)

TVA HEK cells cultured in 96-well plates were prepared for immunofluorescence (IF) via methanol fixation and permeabilisation as described above. Fixed HEKs were blocked for 30 minutes at room temperature in HEK IF block (1x TBS containing 5% goat serum, 0.2x fish gelatin, 0.3% Triton X-100 and 0.01% sodium azide), after which they were incubated overnight at 4°C in primary antibody solutions made up in HEK IF block at the following concentrations: Mouse anti-2A peptide (MABS2005, Merck) 1:1000; Rabbit SP70 anti-tau (MA5-16404) 1:1000.

The following day, primary antibody was aspirated, and cells washed with PBS. Secondary antibody solutions were made up in HEK IF block at the following concentrations: goat anti-mouse Alexa Fluor 647 (Invitrogen, A32728) 1:500; goat anti-rabbit Alexa Fluor 568 (Invitrogen, A11011) 1:500. HEKs were incubated with secondary antibody for 1 hour at room temperature in the dark, after which antibody was removed and neurons were washed with PBS. Cells were left in PBS for fluorescence imaging using an Eclipse Ti2 Microscope.

Molecular characterization of tau/H3-2 and tau/ F8-2$_{\mathbf{S54L+T127A}}$ interactions

Production and purification of the recombinant tau 2N4R and Nb H3-2 and F8-2 $_{S54L+T127A}$ and NMR analysis of tau-H3-2 complex have been performed according to (*18*). Affinity measurements were performed on a BIAcore T200 optical biosensor instrument (Cytiva), according to (*18*). Briefly, biotinylated-tau was injected on a streptavidin SA sensorchip in HBS-EP + buffer (Cytiva), at a flow-rate of 30 µL/min, until the total amount of captured tau reached 500 resonance units (RUs). Nb H3-2 and F8-2$_{\mathbf{S54L+T127A}}$ was injected sequentially with increasing concentrations ranging between 0.125 and 2 mM in a single cycle, with regeneration (three successive washes of 1M NaCl). Single-Cycle Kinetics (SCK) analysis was performed to determine association k_{on} and dissociation k_{off} rate constants by curve fitting of the sensorgrams using the 1:1 Langmuir model of interaction of the BIAevaluation software 2.0 (Cytiva). Dissociation equilibrium constants (K_D) were calculated as k_{off}/k_{on}.

Quantification of tau-venus degradation in TVA/TVS cells following AAV1/2 transduction

20,000 TVA or TVS cells were plated per well of a 96-well plate in FBS free DMEM and transduced with 100,000vg/cell AAV1/2 encoding either R-Nb$_{vhhGFP4}$ or TRIM21 RING or

vhhGFP4 only controls. Cells were longitudinally imaged in situ in an Incucyte S3 system (Sartorius), with images being acquired once every hour.

Validation of VCP inhibition via NMS-873

HEK293 cells were plated in a 24-well plate at 100,000 cells/well. After 24 hours, media was changed to complete DMEM plus 5µM small molecule inhibitor of VCP, NMS-873 or DMSO control. AdV5-GFP was mixed with hIgG1 9C12 antibody with a final concentration of 20ng/ml for 1 hour at RT to allow complex formation. Following 2 hours of pretreatment with drugs or DMSO, 20µl of virus:antibody complexes were added per 500 µl of DMEM per well and incubated for 6 hours at 37ºC, before full media change with complete DMEM. The following day after infection, cells were collected by trypsinisation and GFP infection was analysed via flow cytometry using a Cytoflex (Beckman Coulter) machine. Fold neutralisation was calculated by dividing % infection virus only by % infection with respective antibody concentration.

Diagram Creation

Diagrams presented in figures 1A-D were created with Biorender.com

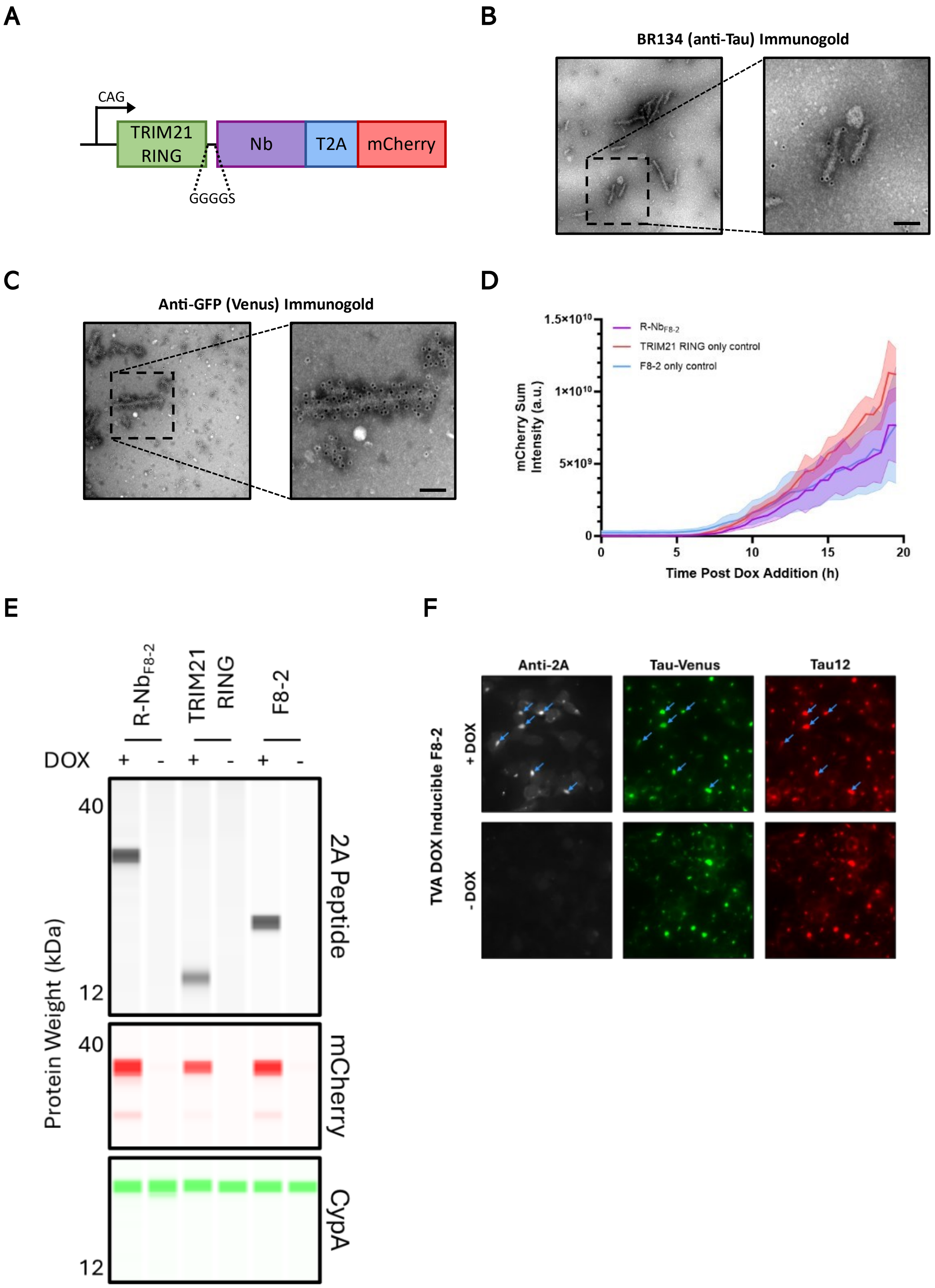
A
CAG
TRIM21 RING
Nb
T2A
mCherry
GGGGS
B
BR134 (anti-Tau) Immunogold
C
Anti-GFP (Venus) Immunogold
D
1.5×10¹⁰
1×10¹⁰
5×10⁹
0
R-Nb F8-2
TRIM21 RING only control
F8-2 only control
mCherry Sum Intensity (a.u.)
0
5
10
15
20
Time Post Dox Addition (h)
E
R-Nb F8-2
TRIM21 RING
F8-2
DOX
+
-
+
-
+
-
40
12
40
12
Protein Weight (kDa)
2A Peptide
mCherry
CypA
F
Anti-2A
Tau-Venus
Tau12
TVA DOX Inducible F8-2
+ DOX
- DOX

Fig. S1. R-Nb design, TVA fibril characterisation, and DOX inducible expression of constructs in TVA cells.

(**A**) Schematic of R-Nb constructs consisting of an N-terminal TRIM21 RING domain (residues 1-85) fused to a nanobody via a flexible GGGGS linker. A self-cleaving T2A-mCherry fluorescent tag was included downstream of the R-Nb construct, with expression being driven by the ubiquitous CAG promoter. (**B**) Immunogold electron microscopy images labelling fibrillar aggregates extracted from TVA cells with the BR134 anti-tau antibody, or (**C**) an anti-GFP antibody. Scale bars, 100nm. (**D**) Sum mCherry intensity from TVA cells expressing either DOX inducible R-Nb_{F8-2}, TRIM21 RING or F8-2, following DOX addition. (**E**) Immunoblot of lysates from the same TVA cells +/- DOX addition for 15 hours, probing for mCherry, CypA (loading control) and the N-terminal product (either R-Nb_{F8-2}, TRIM21 RING, or F8-2) via an anti-2A peptide antibody. (**F**) IF images from TVA cells expressing DOX inducible F8-2 nanobody (15 hours post DOX addition) demonstrating colocalisation (blue arrows) of F8-2 (2A antibody signal) with aggregated tau venus and Tau12 antibody staining. Shaded areas represent mean ± SD. (D) n = 8 biological replicates per condition.

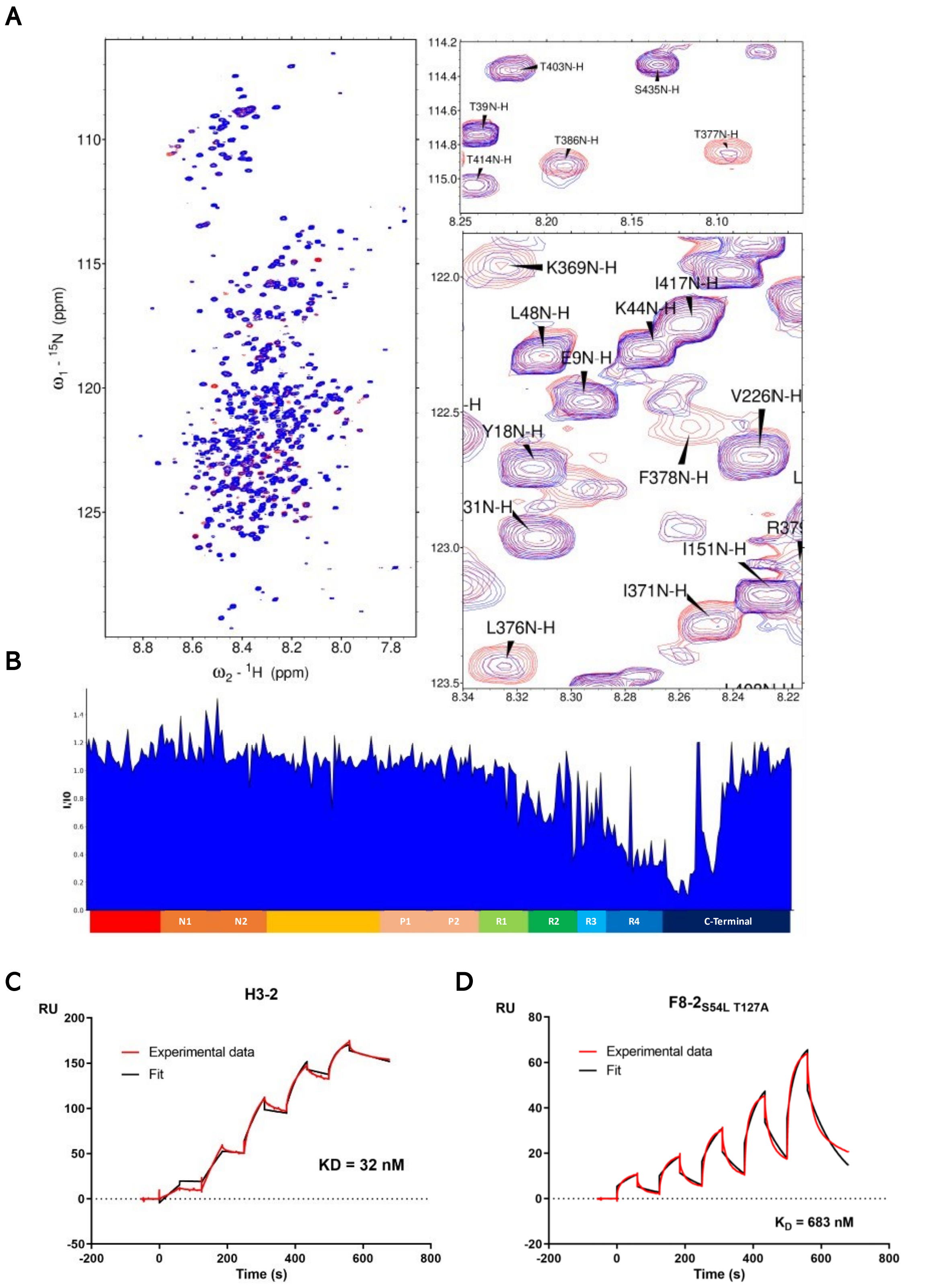

VHH	k_{on} ($M^{-1}.s^{-1}$)*10^{+2}	k_{off} (s^{-1})*10^{-3}	KD (nM)
H3-2	207 ± 2,1	0,7 ± 0,02	32 ± 1,2

VHH	k_{on} ($M^{-1}.s^{-1}$)*10^{+2}	k_{off} (s^{-1})*10^{-3}	KD (nM)
F8-2$_{S54L\ T127A}$	143,7 ± 1,47	9,8 ± 0,05	683 ± 8

Fig. S2. Determination of H3-2 nanobody epitope and H3-2/F8-2$_{S54L+T127A}$ affinities.

(**A**) Overlay of ^{1}H, ^{15}N HSQC two-dimensional spectra and enlargements of free 2N4R tau (in red) or 2N4R tau mixed at equimolar ratio with non-labeled H3-2 nanobody (superimposed in blue) (n = 1). In the spectrum of tau in the presence of H3-2, multiple resonances are broadened beyond detection compared with the tau control spectrum. (**B**) Normalized NMR intensities (I/I0) along the tau sequence with (I0) and (I) corresponding to the resonance intensity when tau is free in solution or mixed with equimolar quantity of H3-2 (I), respectively. The normalized intensity ratios (I/I0) plot allowed the identification of the tau C-terminus domain as the target of H3-2 interaction. N1 and N2 are two alternatively-spliced regions in the N-terminal domain (1–163), the proline-rich domain is subdivided in P1 and P2 regions, the MTBD consists of four partially repeated regions, R1 to R4. (**C**) Sensorgrams (reference subtracted data) of single cycle kinetics analysis performed on immobilized biotinylated tau, with five injections of H3-2 or (**D**) F8-$2_{S54L+T127A}$ at 0.125 µM, 0.25 µM, 0.5 µM, 1 µM, and 2 µM (n = 1). Dissociation equilibrium constant (K_D) was calculated from the ratio of off-rate and on-rate kinetic constants k_{off}/k_{on}. K_{on} and k_{off} values are included in the table. Black lines correspond to the fitted curves, red lines to the measurements.

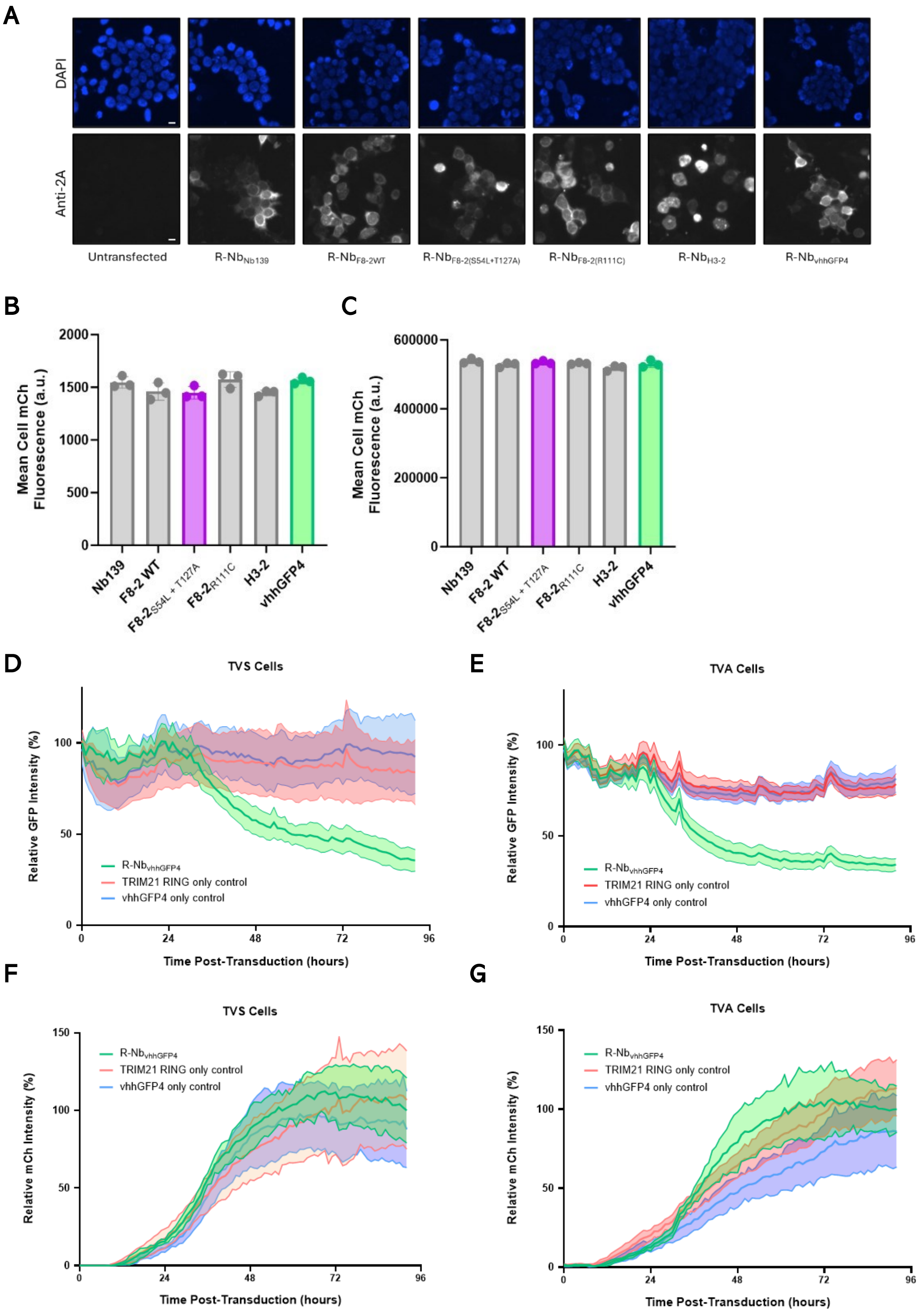
A
DAPI
Anti-2A
Untransfected
R-NbNb139
R-NbF8-2WT
R-NbF8-2(S54L+T127A)
R-NbF8-2(R111C)
R-NbH3-2
R-NbvhhGFP4
B
Mean Cell mCh Fluorescence (a.u.)
Nb139
F8-2 WT
F8-2S54L + T127A
F8-2R111C
H3-2
vhhGFP4
C
D
TVS Cells
Relative GFP Intensity (%)
R-NbvhhGFP4
TRIM21 RING only control
vhhGFP4 only control
Time Post-Transduction (hours)
E
TVA Cells
F
Relative mCh Intensity (%)
G

Fig. S3. Expression of R-Nb constructs in HEK293, TVA and TVS cells, and tau-venus degradation kinetics.

(**A**) Representative IF images from HEK293 cells transfected with R-Nb constructs, stained for R-Nb protein via an anti-2A antibody and for nuclei with DAPI. (**B**) R-Nb expression in transfected and analysed TVA and (**C**) TVS cells, quantified by either fluorescent microscopy or flow cytometry respectively as mean cell mCherry fluorescence. (**D**) GFP intensity (normalised to cell count and plotted as % signal compared to zero-hour timepoint) of TVS cells or (**E**) TVA cells (GFP intensity normalised against confluence and plotted as % signal compared to zero-hour timepoint), over time following transduction with AAV1/2 encoding either R-Nb$_{vhhGFP4}$ or TRIM21 RING or vhhGFP4 only controls. (**F**) mCherry positive cell count (normalised to total cell count and plotted as % signal compared to zero-hour timepoint) from TVS cells or (**G**) TVA cells (mCherry positive cell count normalised against confluence and plotted as % signal compared to zero-hour timepoint), over time following transduction with AAV1/2 encoding either R-Nb$_{vhhGFP4}$ or TRIM21 RING or vhhGFP4 only controls. Shaded areas represent mean ± SD. (B) and (C) n = 3 biological repeats per condition. (D-G) n = 5 technical replicates per condition.

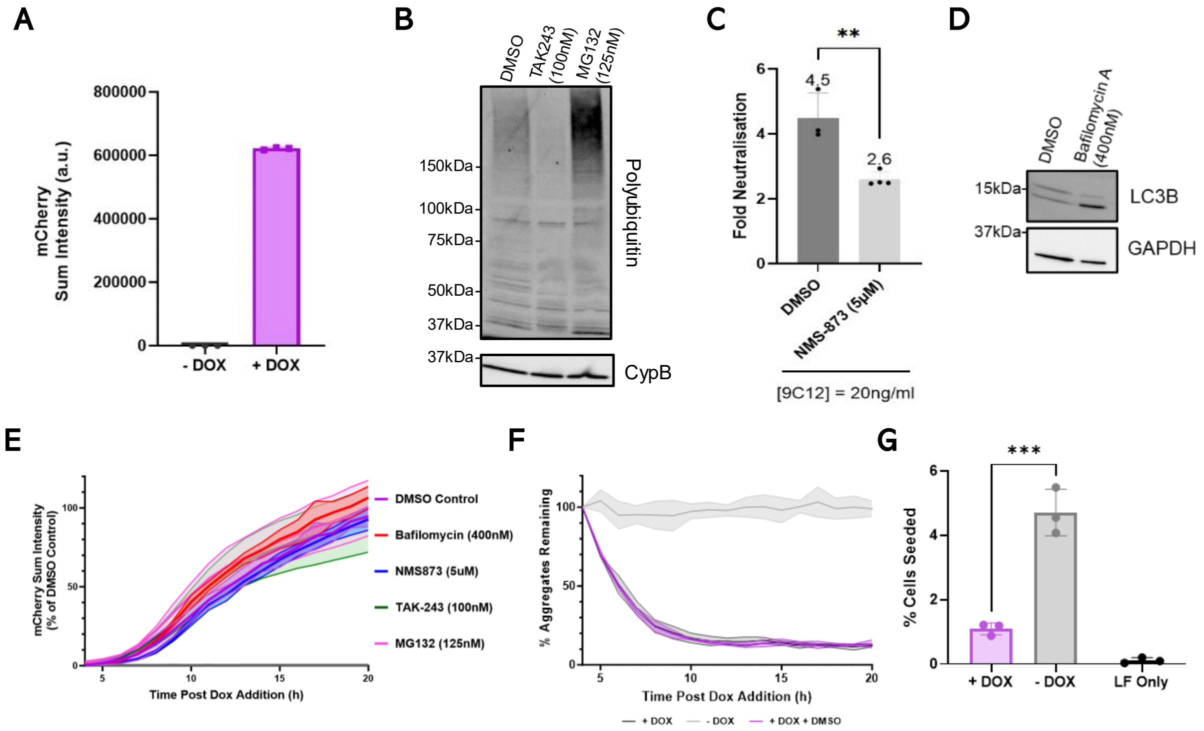

Fig. S4. Expression of R-Nb in TVS Cells, Validation and Effects of Pharmacological Inhibition on R-Nb Expression, and Supernatant Secondary Seeding

(**A**) Integrated mCherry intensity from TVS cells expressing DOX inducible R-Nb$_{F8-2}$ 24 hours after treatment with, or without, DOX, quantified via fluorescent microscopy. (**B**) Western blot of TVA cells treated with DOX and DMSO (solvent control), TAK243 (E1 inhibitor) or MG132 (proteasome inhibitor) for 10 hours. Samples were blotted for ubiquitin to validate inhibitor function, and for CypB as a loading control. (**C**) VCP is essential for antibody/TRIM21 mediated virus neutralization (*15*). The ability of NMS-873 to inhibit VCP was therefore assessed via a viral neutralization assay in which adenovirus encoding GFP was neutralised by the anti-adenovirus antibody 9C12. (**D**) Western blot of TVA cells treated with DOX and DMSO or Bafilomycin A for 10 hours, probed for increased LC3B to confirm autophagy inhibition, and GAPDH as a loading control. (**E**) Sum mCherry intensity from DOX inducible R-Nb$_{F8-2}$ TVA cells following addition of DOX and various small molecule inhibitors, quantified via fluorescent microscopy. (**F**) Quantification of tau-venus aggregates from live cell imaging of TVA cells expressing DOX inducible R-Nb$_{F8-2}$ treated with and without DMSO at the highest concentration used as solvent for small molecule inhibitors (0.5%). Quantified via live cell microscopy. (**G**) Supernatants from TVA cells treated with, or without, DOX for 72 hours to induce expression of R-Nb$_{F8-2}$ were introduced to TVS cells via Lipofectamine. Seeded aggregation of soluble tau-venus was quantified 72 hours later via fluorescent microscopy. TVS cells were treated with lipofectamine (LF) only as a negative control. Error bars and shaded areas represent mean ± SD. (A), (E), (F) and (G) n = 3 biological replicates per condition. (C) n = 4 biological replicates per condition. (C) unpaired *t* test. (G) one-way analysis of variance (ANOVA) with Tukey's correction for multiple comparisons. $^{**}P < 0.01$; $^{***}P < 0.001$.

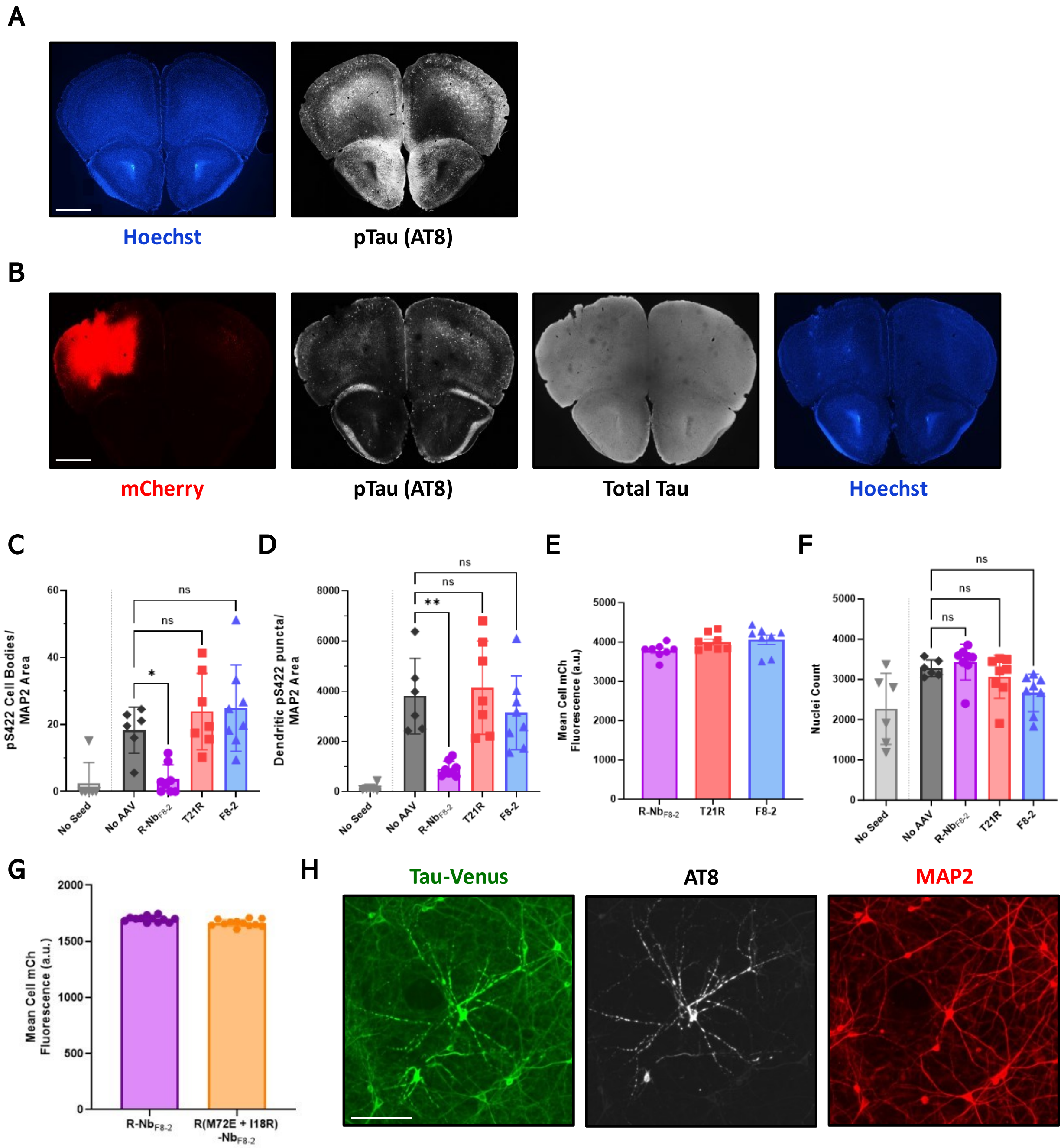

Fig. S5. Reduction of tau pathology in the aged mouse brain and primary neurons via R-Nb$_{F8-2}$.

(**A**) Representative IF images from frontal cortex of 5.5-month-old Tg2541 mouse. Stained for total nuclei with Hoechst and phosphorylated tau aggregates with AT8. Scale bar, 1mm (**B**) Representative IF images 10 days post-stereotaxic injection of AAV1/2-CAG-R-Nb_{F8-2}-T2A-mCherry into the frontal cortex of a 5.5-month-old mouse. Two injections of $2x10^9$ GC were used. pTau and total tau were detected using AT8 and DAKO antibodies respectively. Scale bar, 1mm. (**C**) Quantification of pS422 positive cell bodies and (**D**) dendritic puncta normalised against MAP2 coverage from IF images. Seeded neurons were pre-treated with AAV1/2 encoding R-Nb_{F8-2}, TRIM21 RING (T21R) or F8-2 only. (**E**) Mean cell mCherry fluorescence of primary neuron cultures treated with AAV1/2-hSyn encoding either R-Nb_{F8-2}, TRIM21 RING, or F8-2 with a T2A-mCherry reporter. (**F**) Mean number of nuclei per well of unseeded and tau seeded primary neurons treated with or without the same AAV1/2-hSyn vectors. (**G**) Mean cell mCherry fluorescence of primary neuron cultures treated with AAV1/2-hSyn encoding either active or catalytically dead (M72E+I18R RING mutations) R-Nb_{F8-2} with a T2A-mCherry reporter. (**H**) Representative images from primary neuron cultures prepared from the cortices of neonatal wild-type C57BL/6 mouse pups transduced with AAV1/2-hSyn-0N4R P301S tau-venus (20,000 GC/cell) for 12 days, followed by seeding with heparin assembled P301S tau assemblies for 7 days. Tau-venus was detected with an anti-GFP antibody, with phosphorylated tau aggregates detected using the AT8 antibody and neurons identified via MAP2 staining. Scale bar, 100µm. Error bars represent mean ± SD. (C-F) each point represents the average of two technical repeats from n = 6 to 8 biological repeats. (G) each point represents one of n = 12 biological replicates. (C, D and F) ANOVA with Tukey's correction for multiple comparisons. ns, not significant; *$P < 0.05$; **$P < 0.01$.

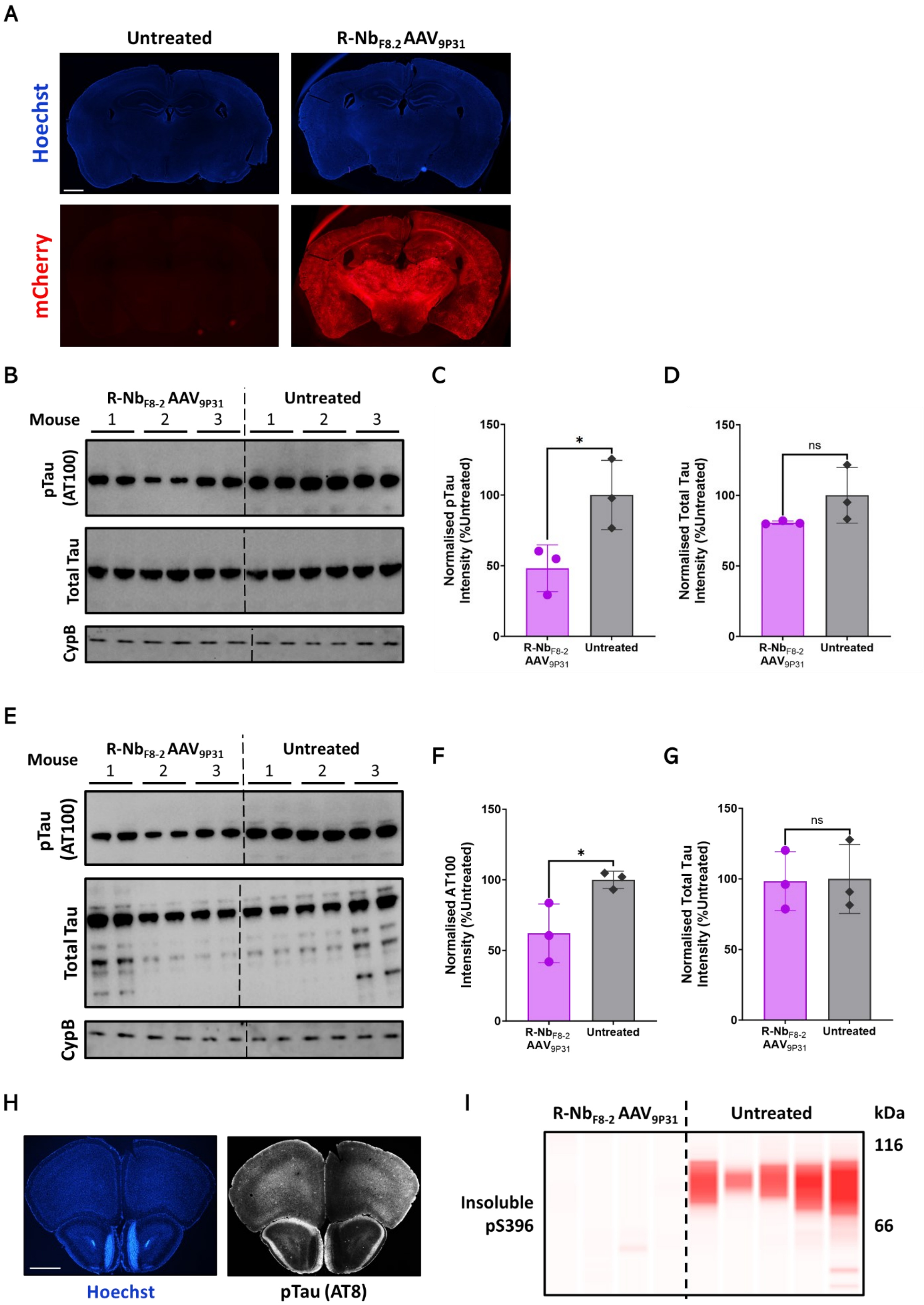
A
Untreated
R-Nb F8.2 AAV 9P31
Hoechst
mCherry
B
R-Nb F8-2 AAV 9P31
Untreated
Mouse
1 2 3 1 2 3
pTau (AT100)
Total Tau
CypB
C
Normalised pTau Intensity (%Untreated)
*
R-Nb F8-2 AAV 9P31
Untreated
D
Normalised Total Tau Intensity (%Untreated)
ns
E
F
Normalised AT100 Intensity (%Untreated)
G
H
Hoechst
pTau (AT8)
I
kDa
116
66
Insoluble pS396

Fig. S6. AAV_{9P31} transduction efficiency and blots from Tg2541 mice treated with AAV_{9P31} R-$Nb_{F8\text{-}2}$ for 10 days or 2 months.

(**A**) Representative fluorescent microscopy images of brain sections from an adult mouse 2 weeks after tail vein injection with $5x10^{11}$ CG of AAV_{9P31} R-$Nb_{F8\text{-}2}$ (with T2A-mCherry reporter). Scale bar, 1mm (**B**) Western blot on whole brain lysates from 5.5-month-old mice treated with $1x10^{11}$ GC AAV_{9P31} R-$Nb_{F8\text{-}2}$ for 10 days, compared to untreated mice. Samples from each mouse were run in duplicate and probed for pTau (AT100), total tau (BR134), and CypB as a loading control. (**C**) Quantification of AT100 pTau and (**D**) total tau (BR134) band intensity in samples from AAV_{9P31} R-$Nb_{F8\text{-}2}$ treated, and untreated mice. (**E**) Spinal cord lysates from the same mice were similarly probed for pTau (AT100) and total tau (BR134), with band intensities quantified in (**F**) and (**G**) respectively. (**H**) Representative IF images from frontal cortex of 4-month-old Tg2541 mouse. Stained for total nuclei with Hoechst and phosphorylated tau aggregates with AT8. Scale bar, 1mm. (**I**) Capillary based immunoblot of sarkosyl insoluble fractions from brains of aged mice treated with or without $1x10^{11}$ GC R-$Nb_{F8\text{-}2}$ AAV_{9P31} for two months, probed for pTau phosphorylated on Ser396. Each lane represents an individual mouse. Error bars represent mean ± SD. (C), (D), (F) and (G) each point represents the average intensity of two technical replicates from one mouse, normalised to CypB signal, plotted as percentage intensity compared to untreated mice. n = 3 mice per condition. Unpaired *t* test, ns, not significant; $*P < 0.05$.

Video S1. Live cell fluorescent microscopy of TVA DOX inducible R-$Nb_{F8\text{-}2}$ cells.

Images were captured every 20 minutes for 15 hours, beginning 4 hours post-DOX addition. Tau venus aggregates were visualised in the green channel, with the red channel detecting mCherry (R-Nb_{F8-2} expression reporter). Scale bar, 20µm.

Video S2. Live cell fluorescent microscopy of tau seeded WT neurons expressing tau-venus, transduced with R-Nb_{F8-2} AAV1/2.

Images were captured every three hours for 7 days following R-Nb_{F8-2} addition. Tau venus aggregates were visualised in the green channel, with the red channel detecting mCherry (R-Nb_{F8-2} expression reporter). Scale bar, 20µm.
